# A Network Approach to Psychopathology: New Insights into Clinical Longitudinal Data

**DOI:** 10.1371/journal.pone.0060188

**Published:** 2013-04-04

**Authors:** Laura F. Bringmann, Nathalie Vissers, Marieke Wichers, Nicole Geschwind, Peter Kuppens, Frenk Peeters, Denny Borsboom, Francis Tuerlinckx

**Affiliations:** 1 Department of Psychology, University of Leuven, Leuven, Belgium; 2 Department of Psychiatry and Neuropsychology, Maastricht University, Maastricht, The Netherlands; 3 Department of Clinical Psychological Science, Maastricht University, Maastricht, The Netherlands; 4 Department of Psychology, University of Amsterdam, Amsterdam, The Netherlands; University of South Florida, United States of America

## Abstract

In the network approach to psychopathology, disorders are conceptualized as networks of mutually interacting symptoms (e.g., depressed mood) and transdiagnostic factors (e.g., rumination). This suggests that it is necessary to study how symptoms dynamically interact over time in a network architecture. In the present paper, we show how such an architecture can be constructed on the basis of time-series data obtained through Experience Sampling Methodology (ESM). The proposed methodology determines the parameters for the interaction between nodes in the network by estimating a multilevel vector autoregression (VAR) model on the data. The methodology allows combining between-subject and within-subject information in a multilevel framework. The resulting network architecture can subsequently be analyzed through network analysis techniques. In the present study, we apply the method to a set of items that assess mood-related factors. We show that the analysis generates a plausible and replicable network architecture, the structure of which is related to variables such as neuroticism; that is, for subjects who score high on neuroticism, worrying plays a more central role in the network. Implications and extensions of the methodology are discussed.

## Introduction

Theoretical considerations and empirical evidence in psychology point towards a network perspective, in which psychological constructs are conceptualized as networks of interacting components instead of measurements of a latent construct, as hypothesized in traditional perspectives [1]–[6]. From this perspective, mental disorders are understood as *networks of interacting symptoms* [3] that form *mechanistic property clusters* [7]: sets of causally intertwined properties that need not share one fundamental underlying cause. By focusing on the interaction between symptoms, the network approach naturally captures the fact that symptoms of psychopathology co-evolve dynamically [8]: if one symptom arises (e.g., insomnia), that symptom can cause other symptoms to arise as well (e.g., concentration problems [3]).

Such patterns of symptom interaction are likely to vary across individuals. For instance, some people have a higher degree of emotional variability than others, and such differences are known to be related to personality traits, such as neuroticism [9]. Likewise, some people may feature stronger connections between sleep deprivation and affect, such that a night of bad sleep quickly leads to depressed mood, whereas others may be more resilient (see e.g., [10]). By focusing on patterns of symptom dynamics, the network approach may potentially yield important insights into how the dynamics of psychopathology relate to intra- and inter-individual differences. Despite the fact that the network perspective is highly suggestive in this respect, techniques to actually empirically chart differences in the dynamical structure of individuals’ symptom dynamics have so far been lacking. In this paper, we present a methodology suited for this task and we apply this methodology to data of individuals with residual depressive symptoms [11] to illustrate its potential use in psychopathology research.

The natural starting point for the study of symptom network dynamics lies in the analysis of symptoms measured over different time points. Such time series data have recently become available due to the rising popularity of data collection approaches using the Experience Sampling Method (ESM), where data about the experiences and affect of participants in their daily life are collected repeatedly over time [12]–[14]. However, current statistical tools for inferring networks from empirical data, as they have been developed and applied mostly in systems biology (see e.g., [15]) and neuroscience (see e.g., [16], [17]), are not optimally suited for data from ESM studies, for several reasons. First, ESM studies do not feature very long time series on a single system (i.e., the number of time points per subject is limited), which hampers the application of typical time series modeling techniques (e.g., [18], [19]). Secondly, ESM data are hierarchically structured because several persons are measured repeatedly leading to measurements that are clustered within persons [20]. This hierarchical structure necessitates the use of separate models for each individual. In combination with the relatively short time series, this leads to unstable results when traditional network models are applied.

In the present article, we demonstrate a statistical method that is tailored to extract network structures from ESM data. We present a multilevel approach to vector autoregressive (VAR) modeling that optimally utilizes the nested structure that typically arises in ESM protocols. This approach is applied to data from an ESM study with a sample of people who feature residual depressive symptoms after a depressive episode (see [11]), and validated in a normal sample. This paper presents the first glimpses of the dynamic weighted network architecture of psychopathology, and develops a methodology that yields new possibilities to analyze and understand the structure of disorders.

The outline of the paper is as follows: first, we elaborate on the ESM study used for the analysis and introduce the methodology, the multilevel-VAR method. Second, we explain how a network can be inferred from the data by estimating the average connection strengths between symptoms or variables of interest. Third, we show how the multilevel-VAR method provides information about inter-individual differences in addition to the average network. Fourth, we discuss how network models can be extended with explanatory variables, and how the networks as such can be further analyzed through local and global analyses. In the fifth section, we show how much of the main results can be replicated using an independent dataset that serves as cross-validation. The software code (in *R*; [21]) and data necessary to perform the analyses that result in the main figures reported in this article are included in Appendix S1 and Data S1 respectively.

## Method

### Data

We inferred a network structure of six items from an ESM study [11]. The ESM study followed 129 participants with residual depressive symptoms over the course of 12 days, of which the first six days were the baseline period. The following six days took place after 2–3 months, after the participants had been randomly divided into a treatment group (63 participants receiving mindfulness therapy (mean age of 44.6 years and SD = 9.7; 79% female) and a control group (66 participants assigned to a waiting list with a mean age of 43.2 years and SD = 9.5; 73% female). Every day subjects were randomly notified by a beeper in each of ten 90-minute time blocks between 7∶30 am and 10∶30 pm. When signaled, they had to fill out the ESM self-assessment form assessing mood and social context in daily life. This resulted in a maximum of 60 responses per period (baseline or post-baseline). All self-assessments were rated on 7-point Likert scales.

For the purpose of our analysis, we selected a number of items that captured distinctive kinds of mood states. Mood states can be broadly differentiated in terms of their valence (positive/negative) and their degree of arousal (high/low [22]–[26]). We included four items that covered different values of the two factors of the mood space. Regarding positive mood, we chose the items ‘I feel cheerful’ and ‘I feel relaxed’ to represent high and low arousal respectively. For representing negative mood, we chose the items ‘I feel fearful’ and ‘I feel sad’, which capture the subjective experience of high and low arousal respectively [27]–[29]. Furthermore, we included the item ‘worry’ because worrying is thought to play a significant role in emotion regulation, including the onset and maintenance of negative mood [30]–[32]. The sixth item of the network, ‘pleasantness of the event’, concerned the environmental context, and assessed the pleasantness of the most important event that happened between the current and the previous response.

### Introducing Multilevel-VAR

To overcome the difficulties that accompany the analysis of nested longitudinal data we developed a novel combination of VAR (e.g., [18], [33]) and multilevel modeling (e.g., [34]). A VAR model is a multivariate extension of an autoregressive (AR) model [19]. An AR model is typically applied to a repeatedly measured variable obtained from a single subject. In this way, the time dynamics within an individual are modeled. An AR model can be considered as a regression model in which a variable at time point 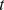 is regressed to a lagged (measured at a previous time point, 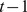) version of that same variable [35]. In VAR the time dynamics is modeled for multiple variables. Thus, variables are regressed on a lagged version of the same variable and all other variables of the multivariate system. By combining the VAR model with a multilevel model, time dynamics can be modeled not only within an individual, but also at group level, since the multilevel model allows the VAR coefficients to differ across individuals. Thus, a combination of both models allows for modeling both individual and population dynamics.

The combination of both modeling approaches has, to the best of our knowledge, not yet extensively been studied or applied in the statistical, psychometric or econometric literature. The methods developed in Lodewyckx, Tuerlinckx, Kuppens, Allen, and Sheeber [36] and Oravecz, Vandekerckhove, and Tuerlinckx [37] have an approach that comes close to what is presented in this paper. However, both methods have a Bayesian and more complex modeling approach and are not easily generalizable to ESM data [36] or can only estimate bivariate symmetric models [37]. Consequently, the specific disadvantages of [36] and [37] make them not directly applicable for network inference as we envision it. The modeling approach of Pe and Kuppens [38] has a similar goal to the method presented in this paper, but makes more approximations (because only bivariate models are used, even though a network of four variables is inferred). Other recent approaches using VAR and/or multilevel can be found in the literature [39]–[43]. However, in the majority of these studies, the dynamic parameters are not treated as random effects but as mere fixed effects (for an exception, see Horváth and Wieringa [43]). In addition, many of these studies do not consider a network approach, nor do they present an accessible way of applying the proposed methodology. In the present paper we present a comprehensive random effects modeling strategy that is optimized to the context of network inference in psychopathology, is implemented in *R* [21], and can be easily passed on to network analysis routines.

### The Population Network

In this section we explain how a population network of the six variables (cheerful, relaxed, sad, worry, fear and event) can be inferred with the multilevel-VAR method. The main goal is to estimate the average connection strengths between all variables in the population. These connection strengths can then be represented in a network. To estimate these connection strengths we apply the multilevel-VAR method to the measured values at baseline of the six variables. For an arbitrarily chosen criterion variable 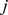 (i.e., cheerful, relaxed, sad, worry, fear or event, for 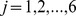, respectively), the model equation is as follows:
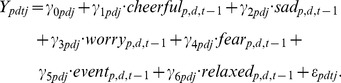
(1)


In our case, 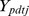 represents the measurement for person *px* (*p* = 1,2,…,129) at day *d* (*d*  =  1,2,…,12) and time 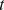 of the 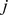-th criterion variable. Equation (1) represents the multiple regression of a single variable at time point 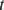 on all other variables at time point 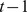. Because there are six variables, there are six such regression equations – one for each variable. At baseline (i.e., at days 1 to 6 before the therapy treatment is applied, such that 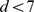), the regression coefficients (i.e., intercept and regression weights) are decomposed as follows:
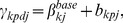
(2)where 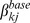 represents the population average effect (fixed effect) at baseline of the lagged variable 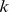
(for 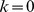, this is the intercept) on the criterion variable 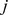, and 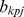 is the person-specific deviation (random effect) of this general effect. In the remainder, person-specific effects will always be denoted in Roman letters.

In order to illustrate our model, let us consider the regression equation for the variable “cheerful”. Because we identify all variables explicitly with their names, we only use the 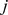-index to distinguish the regression coefficients, but not to identify the variables (hence, the variables carry only three indices, as compared to Equation (1)). At baseline (*d*  =  1,2,…,6), the model reads (not all predictors are explicitly included in the interest of clarity):
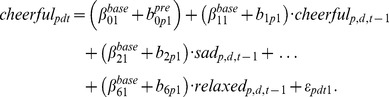



Focusing on the baseline level, we may now construct a 6-by-6 matrix 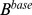 with the fixed effects 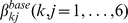. The matrix 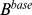 captures the dependence of the 6-dimensional state (i.e., cheerful, sad, worry, fear, event, and relaxed) of a typical individual (i.e., for which 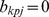) upon the previous 6-dimensional state (all effects at baseline). A specific element 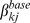 thus expresses the extent to which variable 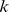 at time point 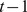 is related to variable 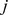 at time 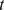, while controlling for all other variables. The elements on the diagonal (i.e., 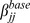) are the autoregressive effects (self-loops), while the off-diagonal elements are the cross-regressive effects (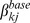; connections between different variables). Note that, in general, 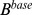 is asymmetric.

The matrix 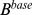 can be viewed as an adjacency matrix [44] of a weighted network. The matrix 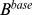 contains the fixed effects of the multilevel-VAR model and represents the lag 1-links between the nodes (i.e., the variables). Thus the matrix 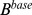 can be thought of as the population average of the network structure. Because we are looking at several specific links, we control for multiple testing by controlling the False Discovery Rate (FDR method; [45]) at 5%. The generated network structure can be visualized through the *R*-package *qgraph* [46]. Only connections that surpass the significance threshold are shown in the visual representation.

Since fitting a multilevel-VAR model directly to the multivariate time series of the participants in the sample is computationally challenging, we approach the problem by breaking up the complicated multivariate problem into a series of easier-to-compute univariate models which are integrated in a second step (i.e., by representing them as a network). This approach can be considered as an instance of the so-called pseudo-likelihood method [47], [48].

By using univariate models, most parameters are estimated directly (e.g., all fixed effects 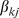 and variances of the error terms 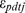). However, some parameters of the model such as the correlations between error terms of the different univariate regression models can only be estimated indirectly in our approach. In Appendix S2, a more elaborate description of the pseudo-likelihood method is given, and it is shown through simulations that point estimates of most directly and indirectly estimated parameters are on average close to the true values. This indicates that the pseudo-likelihood fitting procedure of the multilevel-VAR approach is a feasible alternative to full likelihood fitting procedures. The modeling is carried out using the lme4 package in *R* [49] (see *R*-code in Appendix S1).

### Individual Differences

The multilevel-VAR method provides information about inter-individual differences (random effects) in the network, in addition to the population average network (see Equation (2)). Through the random effects we can construct networks of individual variability and infer a network for each subject of the ESM study separately (see *R*-code in Appendix S1).

In this paper, we take a random effect approach to estimate inter-individual differences, and assume that these person-specific parameters 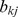 are drawn from a multivariate normal distribution with a zero mean vector and an unstructured covariance matrix (see e.g., [50]; see Equation (2)). Other approaches to deal with inter-individual differences are fixed-effects analysis (i.e., constructing a dummy variable for each subject [51]) and conditional analysis (see e.g., [52]). In the multilevel-VAR method a random-effects approach is taken because it avoids possible problems related to the two previously mentioned approaches. The approach is more parsimonious in terms of number of parameters: instead of having to estimate explicitly all person-specific parameters as in the dummy variable approach, only the variance parameters have to be estimated [53], which at the same time avoids problems of inconsistent estimators [54]. The random-effects approach also allows one to evaluate all effects, in contrast to the conditional analysis approach, in which effects of between-person variables, such as possible therapy effects, cannot be evaluated [55].

To construct a network representing individual variability, we take the estimate of the population standard deviation of the person-specific (random) effects 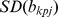. Thus, each connection in the network represents the *SD* of the random effects for that specific connection. Connections in the network that have a large standard deviation represent a high variation of the value (connection strength) of that specific connection over individuals.

In addition, the model in Equations (1) and (2) allows for constructing a network of a single subject. These 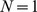 networks are a combination of the individual random effect, which is added to the fixed effect of the relevant link (connection) in the network. For instance, in the individual network of person 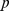 at baseline, the link from node 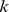 to node 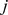 has a value of 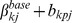 (see Equation (2)).

### Extending the Network Model with Explanatory Variables: Local and Global Network Analyses

As is the case in a standard multilevel analysis, explanatory variables that might explain part of the inter-individual variability can be added (called level-2 variables in standard multilevel terminology, see [34]). In this paper, we present two examples. In the first example, the explanatory variable “therapy-intervention” is added to the standard model. We compare the network of the therapy group with the non-therapy group by comparing specific links in the networks. A therapy effect on the network structure implies a significant three-way interaction. For example, if there is a therapy effect on the link from sad to cheerful, this means that the interaction between the variables therapy (therapy or control), time (pre or post baseline) and sad (ranging from 1 to 7) is significant in the regression model that applies to the variable cheerful, signifying that the effect of feeling sad on feeling cheerful has changed from pre- to post-therapy.

In the second example, we explain variability in individual networks by relating it to covariates; here, neuroticism functions as an example. We present a global network analysis, in which the overall structure of the network is taken into account; these analyses contrast with local network analyses, which compare specific connections across networks. A representative example of such a network analysis is a centrality analysis. We will examine whether the structure of the network regarding centrality changes when the degree of neuroticism changes. This question is approached by looking at differences in the network structure of three different groups: low, mid and high neuroticism.

### Therapy: Local Network Analysis

In order to analyze whether therapy had a significant effect on the network structure we added the variable “therapy-intervention” to the baseline model (see Equation (2); see *R-* code in Appendix S1). Thus, besides reports measured at baseline (i.e., 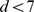), we also added post-baseline measurement instances (i.e., 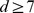). The regression coefficients (for which 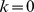 is the intercept and 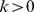 are the regression weights) are now equal to:
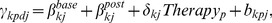
(3)where 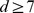 and the term 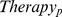 equals to 0 if person 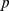 belongs to the control group, and takes value 1 if the person received mindfulness therapy. As can be seen from the equation, 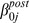 represents the difference between the intercept at baseline and post-baseline for the control group. In general, Equation (3) allows for a difference between the mean of the control and the therapy group, so differences between the two groups post-baseline are accommodated for. A comparison of Equations (2) and (3) shows that the model assumes person-specific deviations from the regression weights to be the same pre- and post-baseline (i.e., persons who deviate in a particular way from the mean structure during baseline will continue to do so post-baseline). This restriction is made for reasons of parsimony. However, for the intercept, the model allows person-specific deviation of the general intercept to be different pre- and post-baseline (therefore the pre-baseline person-specific deviation will be denoted as 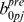 and post-baseline as 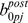).

To illustrate this model, let us consider the regression equations for the variable “cheerful”. The post-baseline 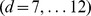 model for the controls becomes
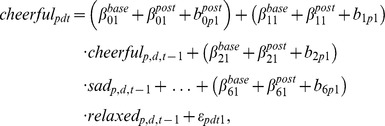
while that for the therapy group equals



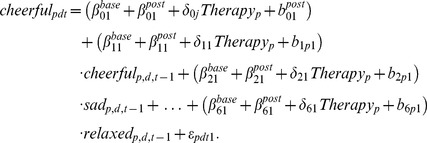
(4)Analogous to the construction of 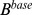, as described in the previous section, we may construct matrices 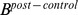 and 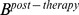, which can be interpreted as network structures that describe the post-intervention behavior of the relevant variables as they apply to control and therapy groups. Through this model we can evaluate the effect of therapy, by looking at the three-way interactions between a predictor variable, the post-baseline indicator and the therapy-indicator (the parameters of interest are 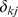 in Equations (3) and (4)). Because we are looking at several specific links, we control for multiple testing. This is done by controlling the False Discovery Rate (FDR method; [45]) at 5%.

### Neuroticism: Global Network Analysis

Important information about a network can be gained by analyzing its global structure, for example by looking at the relative centrality of different nodes. In a centrality analysis, nodes are ordered in terms of the degree to which they occupy a central place in the network. Relevant centrality measures can be constructed in different ways [56]; here, we focus on *betweenness centrality*. Betweenness centrality takes direct and indirect weighted links between the nodes into account. First, for each pair of nodes *x* and *y* (e.g., worry and cheerful), the strongest direct and/or indirect connecting paths from *x* to *y* and from *y* to *x* are determined. Then for each node, it is calculated to which degree the node lies on the shortest path between two other nodes. The more often a node lies on the shortest path between two other nodes, the more the node can funnel and influence the flow in the network, and the higher its betweenness centrality is [56].

To evaluate whether betweenness centrality of the network changes when the degree of neuroticism changes we added the variable neuroticism to the regression model in the same way as the variable therapy was added (see *R*-code in Appendix S1):
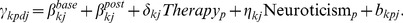
(5)


In this study, neuroticism was assessed with the NEO-FFI scale of neuroticism [57]. In order to be able to deal with possible nonlinear effects of neuroticism on the network structure, the continuous neuroticism measure was subdivided into three groups (based on the three quartiles) resulting in a low, middle and high neuroticism group, corresponding respectively to sum scores 12–34, 35–45, and 46–60 on the NEO-FFI scale. The term Neuroticism*_p_* equals to 0 if person *p* belongs to the low neuroticism group, takes value 1 if the person *p* belongs to the middle neuroticism group and equals to 2 if the person *p* belongs to the high neuroticism group. For reasons of parsimony, we let neuroticism interact with the connection strengths of the baseline network only.

For computing betweenness centrality, we used the *R* package *qgraph* [46]. To assess the uncertainty of betweenness centrality, we used a parametric bootstrap method to construct the distribution of the betweenness statistic under the null hypothesis that the fitted model is correct [58]. To achieve this, we followed a three-step procedure. First, the model as outlined above was fitted to the data. Based on the model estimated from the observed data, we computed the predicted values and residuals for each observation. In the second step, we created 1000 simulated datasets by sampling at random from the vector of residuals and adding the sampled residuals to the predicted values. In the third step, a model was fitted to each of the 1000 simulated datasets, and from the estimated coefficients, the betweenness at baseline for low, mid and high levels of neuroticism was computed. From the distribution of betweenness scores, we calculated the median and the 50% and 95% bootstrap confidence intervals.

The parametric bootstrap procedure is computationally intensive because the model has to be re-estimated for each of the 1000 datasets. This is prohibitively time consuming using a mixed model, and therefore we used a traditional linear model containing only fixed effect coefficients instead of a multilevel model. However, the agreement with point estimates from the linear model is very strong (e.g., the correlation between the fixed parameter estimates from the multilevel model and the linear model is 0.93) and this strong correspondence justifies the approximation step.

### Replication of the Results: A Validation Dataset

In order to test if results found with the multilevel-VAR method could be replicated, we compared the main outcomes of the main dataset with a second validation dataset. The validation data we used was from an ESM study of Kuppens (part of the data are published in [59], [60], [61]). In this ESM study, 97 university students (with a mean age of 19.1 years, SD = 1.3; 63% female) were followed over the course of seven days. The participants had to fill out an ESM self-assessment form assessing mood and social context in daily life 11 times a day. This resulted in a maximum of 77 responses. All self-assessments were rated on scale from 0 to 100. From this dataset, we selected the variables that this set had in common with the variables of the main dataset: cheerful, relaxed, sad, worry and fear. Note that “worry” was assessed slightly differently in the validation study: “How much have you worried since the previous beep” instead of “I am worrying at the moment”. Furthermore, the pleasantness of events was not measured in this study. To increase comparability, networks inferred from these five variables were compared with networks inferred from the five corresponding variables of the main dataset.

First, we inferred a population network containing the five variables cheerful, relaxed, sad, worry and fear for both the main dataset and the validation dataset. Then the connection strength of the links of the main network was correlated with the links of the validation network. The higher the correlation, the better the two inferred networks agree. To assess the correlation, we used both Pearson’s product moment correlation and Spearman’s rank order correlation coefficient. In addition, we assessed to which extent the variances of inter-individual differences are comparable in the two studies. The correlation between the variances of the random effects of the links of both networks was calculated using Spearman’s product moment correlation and Pearson’s rank order correlation coefficient.

In the validation dataset, there is no therapeutic intervention, so the local network analysis could not be replicated. However, neuroticism was measured in the validation set, and thus we applied the global network analysis to the validation set. Hence, we tested whether the centrality of the network changes in the same way in both datasets when the degree of neuroticism varies. Again, we used only the five variables that both sets have in common. In this ESM study, neuroticism was measured with the Dutch version of the Ten Item Personality Inventory [62], [63] with a sum score ranging from 1 to 7. Neuroticism was again subdivided into three groups: a low, middle and high neuroticism group, corresponding respectively to sum scores 1–2, 2.5–4.5, and 5–7 on the TIPI scale.

### Model Assumptions

In order to apply the multilevel-VAR model three assumptions on which the model is built need some further commenting. The first assumption is that we start the clock again at the start of each day as to avoid the day-night problem, which means that we do not use the measurements of yesterday to predict the measurements of today (because a night separates the two days). A night is a relatively large time interval and is psychologically and physiologically qualitatively different from daytime (e.g., [64]). Thus, the first measurement of the day was excluded from analysis. With regard to time it is furthermore assumed that the time intervals between two consecutive measurements are approximately equal. We will come back to both aspects when discussing the results.

Stationarity is a second important assumption inherent to the model. In order for a process to be (weakly) stationary, the mean and variance of the series must stay unchanged over time [65]. Stationarity was tested with the Kwiatkowski-Phillips-Schmidt-Shin (KPSS) test separately for every subject and variable pre and post intervention. The null hypothesis of the KPSS test is that a time series is stationary [66]. Furthermore, a general check was executed to test for a trend, and thus non-stationarity, in the overall data. This was done by comparing the model outlined in the previous section with a model into which a person-specific linear deterministic trend was added (using the beep number as a predictor). For both models, the Bayesian Information Criterion (BIC) was calculated (by summing the separate BICs of the six univariate models). The BIC is a comparative model selection method that takes both the goodness-of-fit of a model and the complexity of the model (as measured by the number of parameters) into account. Models with a large number of parameters are penalized [67]. The model with the lowest BIC is the preferred model.

The specific order of the model is the third assumption. For reasons of parsimony, we present only the results of the baseline models with lag-1 predictors included. However, we also fitted competing models of orders two and three (i.e., with all lags included up to the specified order). In order to keep the problem computationally tractable, we did not allow for random effects on predictors of lags larger than one and in the main dataset we constrained the additional lag effects to be equal at pre- and post-baseline and in control and therapy groups.

## Results

This section is organized as follows. We start by discussing the validity of the stated assumptions (because the validity of the results depends on the veracity of the assumptions). Subsequently, we discuss the population network, individual differences, and the effect of explanatory variables.

### Assumptions

Since a measurement is not allowed to predict the following measurement overnight, we deleted the first measurement of each day. Furthermore the data had to be lagged. Together this led to a reduction in the number of reports included in the analysis: The average number of useable data points went down from an average of 49 to an average of 35 reports for each period (baseline and post-baseline). Regarding the assumption of equally spaced time points, the ESM study, having a quasi-random beeping scheme, violates this assumption. However, the extent of the violation is taken to be small, since the variation in between-measurement points is relatively small with an average of 1.5 hours and a standard deviation of 0.54.

Concerning stationarity, the KPSS test indicated that a vast majority of the data was stationary (about 77%). In addition, the BIC indicated that the models without trend were a better fit to the data (BIC = 172896) than the models with linear trend (BIC = 172995). Thus, overall the data are judged to be sufficiently stationary.

Regarding the lag order, the BIC indicated that the order-3 model fitted best and the order-2 model fitted better than the order-1 model. However, the lag-1 coefficients were very similar across the three models. Since the impulse response functions [18], [68] also did not reveal any substantive effects of interest, which could have warranted a more complex analysis, we proceeded with the order-1 results.

### The Population Network

**Figure 1:**
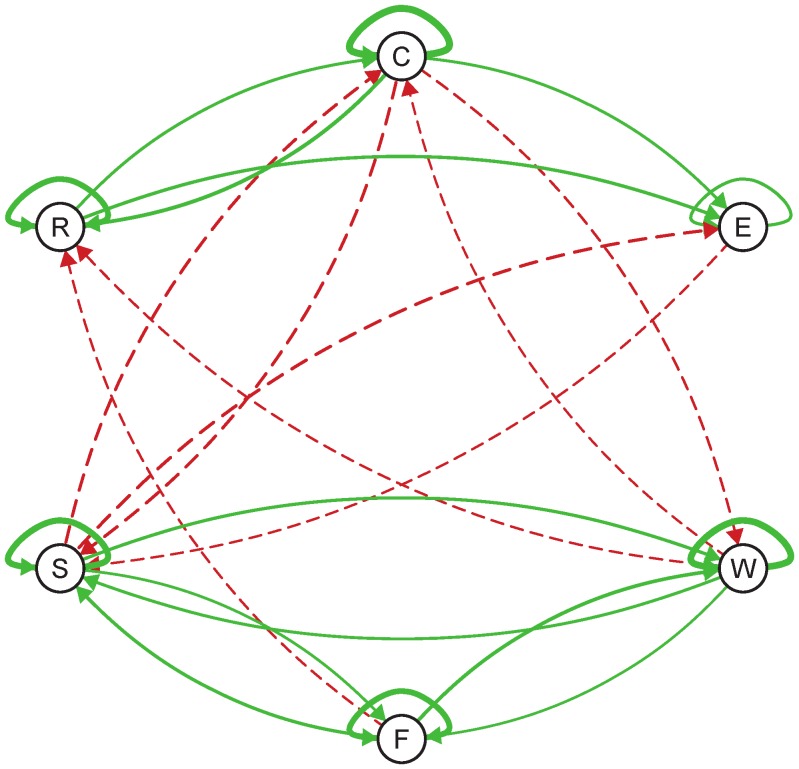
Estimated population network at baseline. The six items are: C = cheerful, E = pleasant event, W = worry, F = fearful, S = sad and R = relaxed. Solid green arrows correspond to positive connections and red dashed arrows to negative connections. Only arrows that surpass the significance threshold are shown (i.e., for which the p-value of the t-statistic is smaller than 0.05). Arrows can be either red, indicating a negative relationship (i.e., 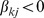), or green, indicating a positive relationship (i.e., 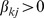). Furthermore, the strength of the relation from item k to item j (i.e., an extremer value for 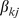) is translated into the thickness of the arrows: the thicker the arrow between two nodes, the stronger the nodes or items are related. Note that item responses can also be predicted from the previous state of the item itself. These arrows are the self-loops in the network.

The inferred population network at baseline is presented in Figure 1 (i.e., the matrix 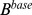). Each variable is represented by a node in the network and relations between items are represented by the weighted arrows (connection strength) between nodes. The arrow from item 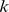 to item 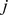 is a visual depiction of the weight 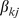, expressing the strength of the relation between item 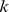 at time 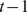 and item 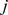 at time 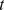. Arrows can be either red, indicating a negative relationship (i.e., 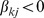), or green, indicating a positive relationship (i.e., 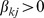). Furthermore, the strength of the relation from item 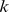 to item 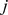 (i.e., a more extreme value of 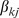) is translated into the thickness of the arrows: the thicker the arrow between two nodes, the stronger the relation between the nodes or items. Note that item responses can also be predicted from the previous state of the item itself. These arrows are the self-loops in the network.

In Figure 1, only arrows that surpass the threshold for significance (i.e., *p*-value of the *t*-statistic is smaller than 0.05) are represented in bold in the network; the non-significant arrows are made transparent. Controlling for multiple testing by controlling the False Discovery Rate (FDR method; [45]) at 5% does not lead to qualitatively or quantitatively different conclusions.

From Figure 1, a few general insights on the dynamical network structure between the six items can be derived. First, in accordance with a dynamical view on emotions, both the positive and the negative items form a cluster representing self-perpetuating cycles in which the components of negative and positive emotions interact (see also [69], [70]). We find that positive or excitatory connections exist among items of the same valence, while negative or inhibitory relationships exist among clusters of mood states of opposite valence (e.g., cheerful, relaxed and pleasant event on the one hand and sad, worry and fearful on the other hand). This is in line with existing theories in affect research [38], [71]–[73].

A second insight from Figure 1 is that the self-loops or autoregressive effects are always positive and they are generally among the strongest connections in the network, indicating that, for instance, the current experience of worry or cheerfulness predicts future feelings of worry or cheerfulness. At a more detailed level, we see that in the baseline model, for example, worry leads to increases in negative affect by enhancing negative moods and inhibiting positive moods. This lines up well with previous findings (e.g., [74]–[76]) and supports the validity of our approach.

### Individual Differences

**Figure 2:**
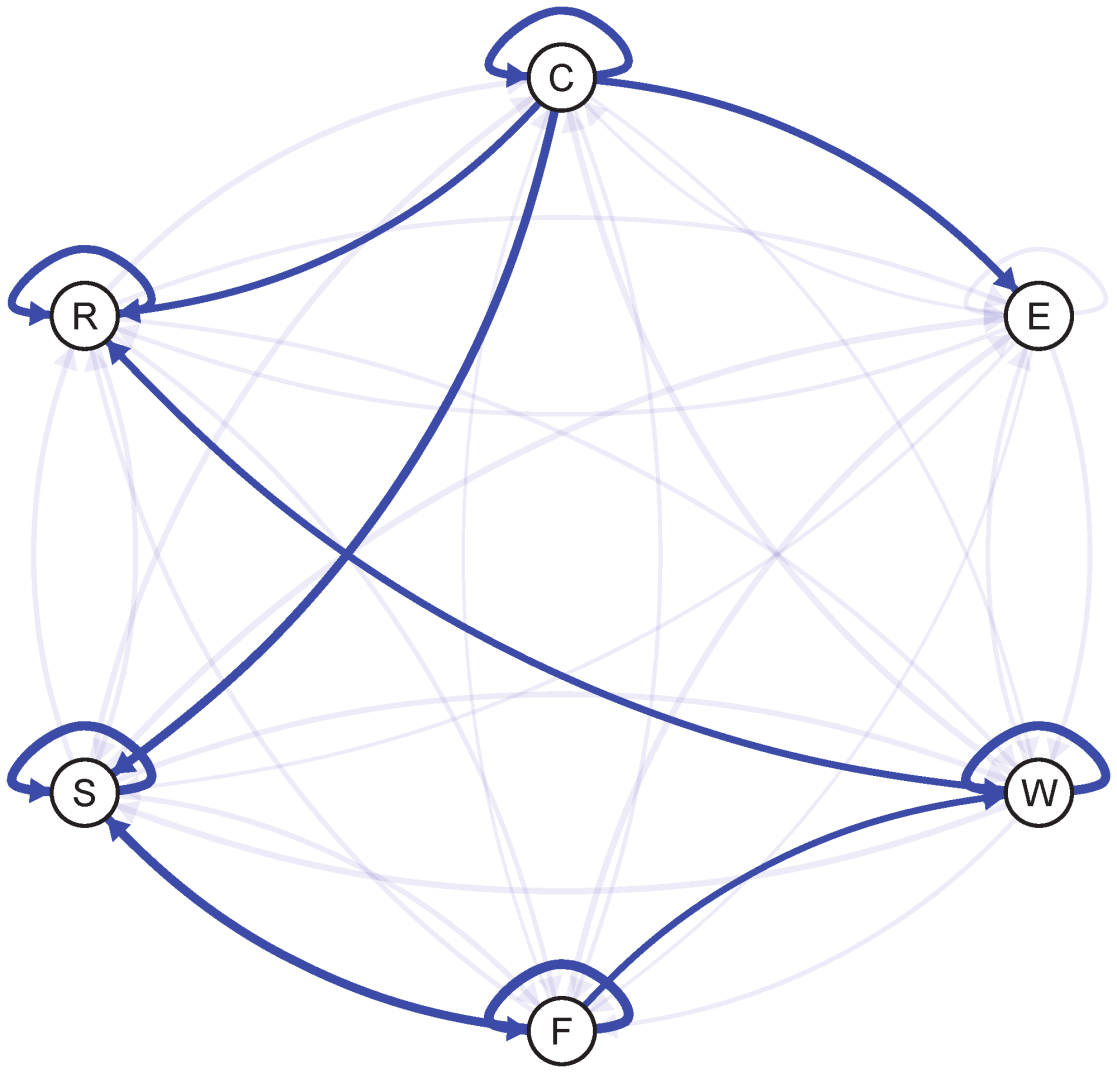
Inter-individual differences of the arrows of the network from Figure 1. The thickness of the arrows is based on the size of the standard deviation of the random effects. To construct the figure, we have put a cutoff of 0.1 on the standard deviation and only the standard deviations above the cutoff are shown with a non-transparent arrow. As the threshold for the standard deviation of the random effects 0.1 was chosen because it represents large inter-individual differences. The average coefficient of the self-loops (i.e., autoregression coefficients) is about 0.2 with a random effects standard deviation of 0.1. Therefore, assuming a normal distribution, the range from 0 to 0.4 represents 95% of the individual self-loop coefficients. With a larger cutoff, such as 0.2, also individuals having negative self-loops would be taken into account. However, more than 95% of the population has a positive self-loop strength.

The multilevel-VAR method also provides information about inter-individual differences (random effects) in the network in addition to the population average network (fixed effects). The links with the largest inter-individual differences are shown in Figure 2.

The arrows in the network now represent the estimated variance of the relevant VAR parameters over individuals. Only arrows containing a 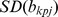 larger than 0.1 are emphasized in Figure 2. For example, the pronounced self-loop on the item ‘worry’ indicates a high individual variability.

**Figure 3:**
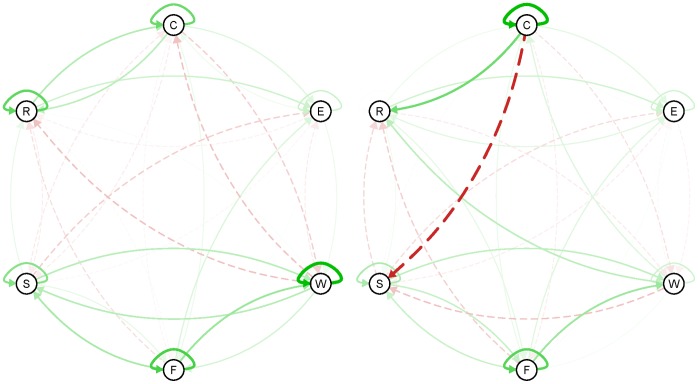
Individual networks (at baseline) of two different persons.

This individual variability can also be immediately observed in the networks of individual subjects. Figure 3 illustrates the individual networks at baseline for two persons. The network on the left has a quite strong self-loop for the item ‘worry’, which means that when this person worries, he or she tends to worry for a longer time. On the other hand, the network of the participant on the right has a weak self-loop for the item ‘worry’, meaning that when this person starts to worry he or she is likely to worry for only a short time. Thus, not only can we verify which arrows have a high inter-individual variability; we can also immediately indicate what these arrows look like in networks that apply to an individual person.

### Therapy: Local Network Analysis

To evaluate the effect of therapy on the local network structure we compared the arrows in the networks of the therapy and the control group. After correcting for multiple testing (using FDR controlled at 5%), none of the arrows indicated a significant effect of therapy on the network structure, meaning that there was no significant three-way interaction between the arrow, the post-baseline indicator and the therapy-indicator. However, this does not imply that there is no effect of therapy at all. First of all, as shown in previous research [11], therapy has an effect on the average levels of some variables, and also in this study we can detect effects of therapy on the mean level of, for instance, cheerfulness. Secondly, the fact that we did not find an effect of therapy on the network structure here could also be due to a lack of power. Correcting for multiple testing always leads to a decrease in power, which can lead to missing an effect on the network structure that is small but still relevant.

### Neuroticism: Global Network Analysis

**Figure 4:**
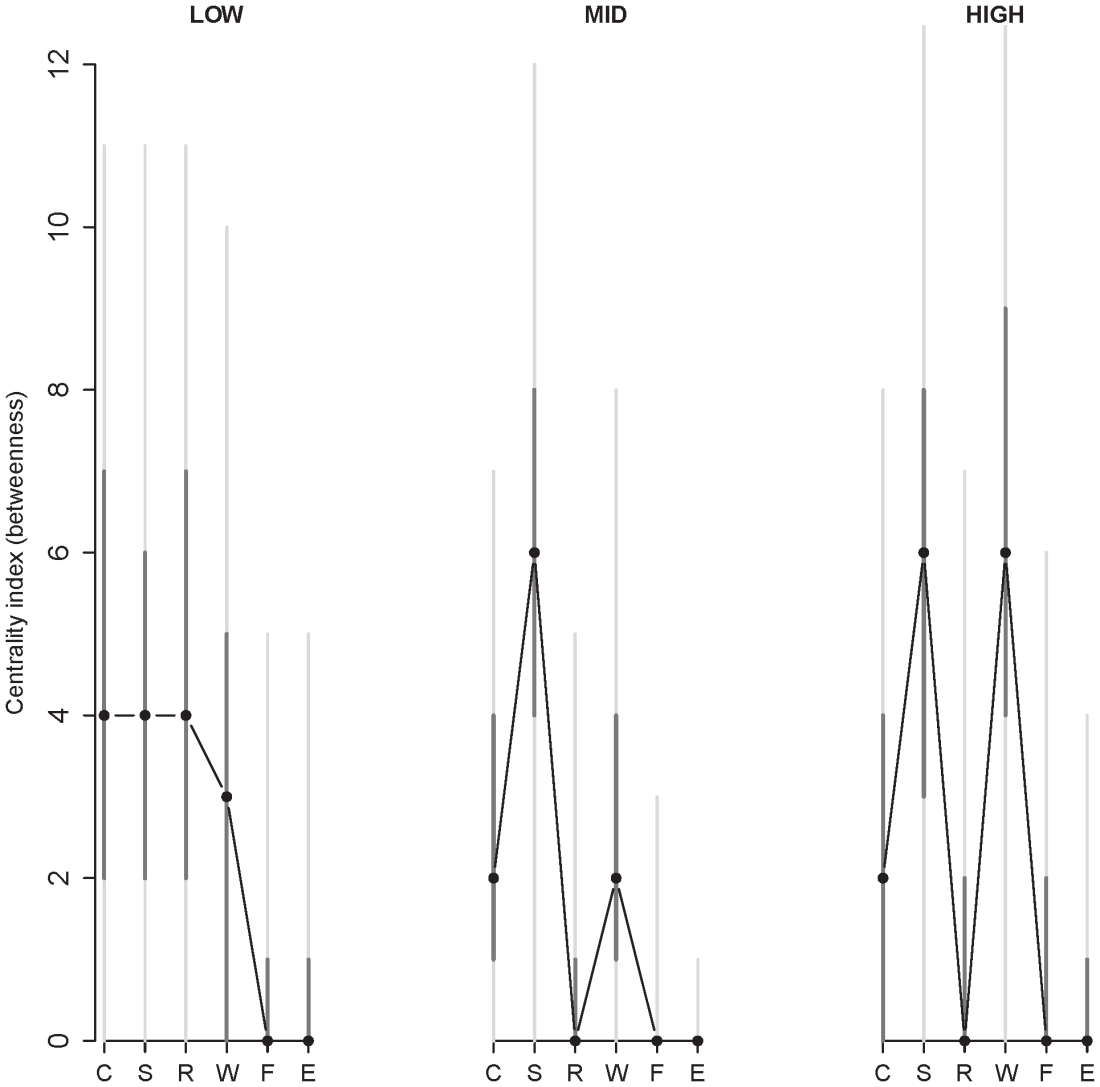
Centrality (betweenness) of each item in the network as a function of level of neuroticism at baseline. Low, mid, and high neuroticism are shown from left to right. The labels of the items are abbreviated by their first letter (C = cheerful, S = sad, R = relaxed, W = worry, F = fearful and E = event). The black dots are the model-based estimate of betweenness, the darkgrey vertical lines represent 50% confidence intervals and the light grey vertical lines represent 95% confidence intervals (as estimated from the bootstrap method). Together, the median, 50% and 95% confidence intervals give information on how the node centrality for every item in all three networks is distributed.

To assess the effect of neuroticism on the global network structure, we tested whether the structure of the network regarding betweenness centrality changes as a function of neuroticism. Figure 4 presents the results of the betweenness analysis for low, middle and high neuroticism at baseline. For every item, the model-based estimate of betweenness is calculated, together with a bootstrap simulated 50% and 95% confidence interval. Plotting both 50% and 95% confidence interval gives an indication of the asymptotic distribution of the estimate.

Although the distributions of the betweenness coefficients are quite wide (as are the associated confidence intervals), the data do suggest some interesting trends. In order to get a good interpretation of the effects of neuroticism on betweenness, it is insightful to look at the effects on the entire betweenness distribution. Whereas the centrality of the nodes fearful and event are low and stable across groups, the positive nodes cheerful and relaxed become less central as neuroticism increases. This is indicated by the distribution, which clearly shifts downwards.

Additionally, the nodes of both sadness and worry increase in centrality as neuroticism increases. Notably, worry has a higher centrality distribution in the high neuroticism group than in the low and mid neuroticism group. That is, worry becomes one of the most central nodes in the high neuroticism group. This result is in line with studies suggesting that worry is an important manifestation of neuroticism [77], and with the idea that worry is a cognitive concomitant of neuroticism [75].

### Replication of the Results: A Validation Dataset

#### Assumptions

The assumptions for applying a multilevel-VAR model were also met in the validation dataset. Excluding the first measurement of each day and lagging the data led to a reduction in the number of reports included in the analysis: The average number of useable data points went down from an average of 60 to an average of 53 reports. In this dataset, the assumption of equally spaced time points was also only slightly violated. The variation in between-measurement points was relatively small with an average of 1.2 hours and a standard deviation of 0.49. Regarding stationarity, the KPSS test indicated that a vast majority of the data was stationary (about 70%). In addition, the BIC indicated that the models without trend (BIC = 202100) were a better fit to the data than the models with linear trend (BIC = 202203), indicating that overall the data is stationary. Because the higher order analyses did not reveal any substantially different conclusions and the aim was to compare the results from the two datasets, we pursued an order-1 analysis.

#### Population network

**Figure 5:**
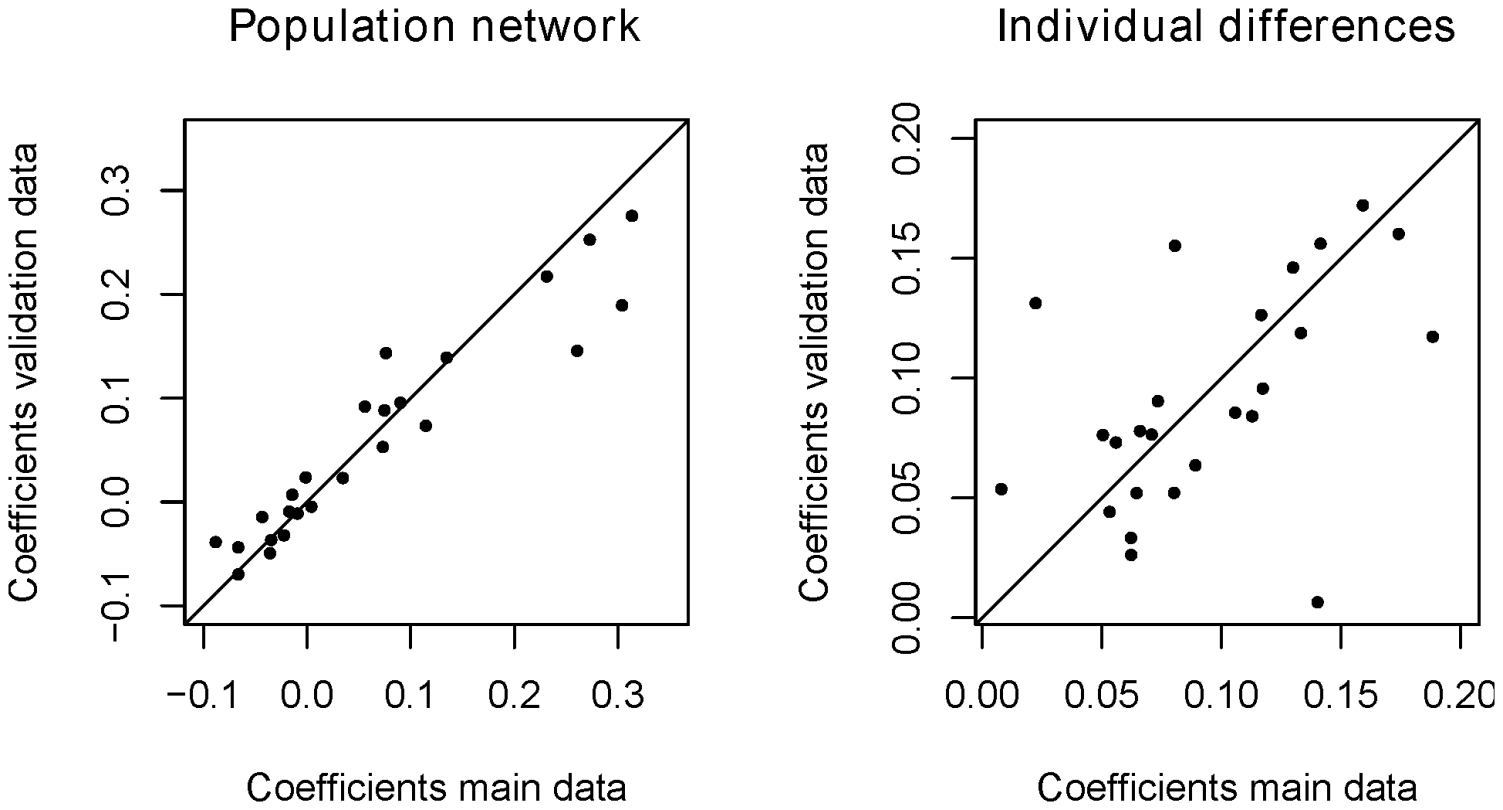
Correspondence between the basis dataset and the validation dataset. Left panel: Representation of the correspondence between the population network coefficients (fixed effects) of the basis dataset (x-axis) and the validation dataset (y-axis). Right panel: Representation of the correspondence between the inter-individual differences (random effects) of the basis data (x-axis) and the validation data (y-axis).

In the left panel of Figure 5, the correlation between the connection strengths of the links of the main population network and the links of the corresponding validation network is shown. The product moment correlation between the connection strengths of the two networks is 0.95 (*p*<0.0001; the rank order correlation is *r = *0.96, *p*<0.0001). This indicates that the population networks between both datasets agree almost perfectly. The networks inferred for the validation data are not shown here, but can be found in Appendix S3.

#### Individual differences

In the right panel of Figure 5 the correlation between the connection strengths of the links of the main inter-individual differences network and the links of the corresponding validation network is shown. The product moment correlation between the connection strengths of the two networks is a sizeable correlation of 0.50 (*p* = 0.01; rank order correlation is *r = *0.56, *p* = 0.004). This indicates that although some links in the inter-individual differences networks differ between the two datasets, the majority of them reflect a similar degree of individual variability. We refer again to Appendix S3 for a visual illustration of the individual differences networks in both datasets.

#### Neuroticism: global network analysis

**Figure 6:**
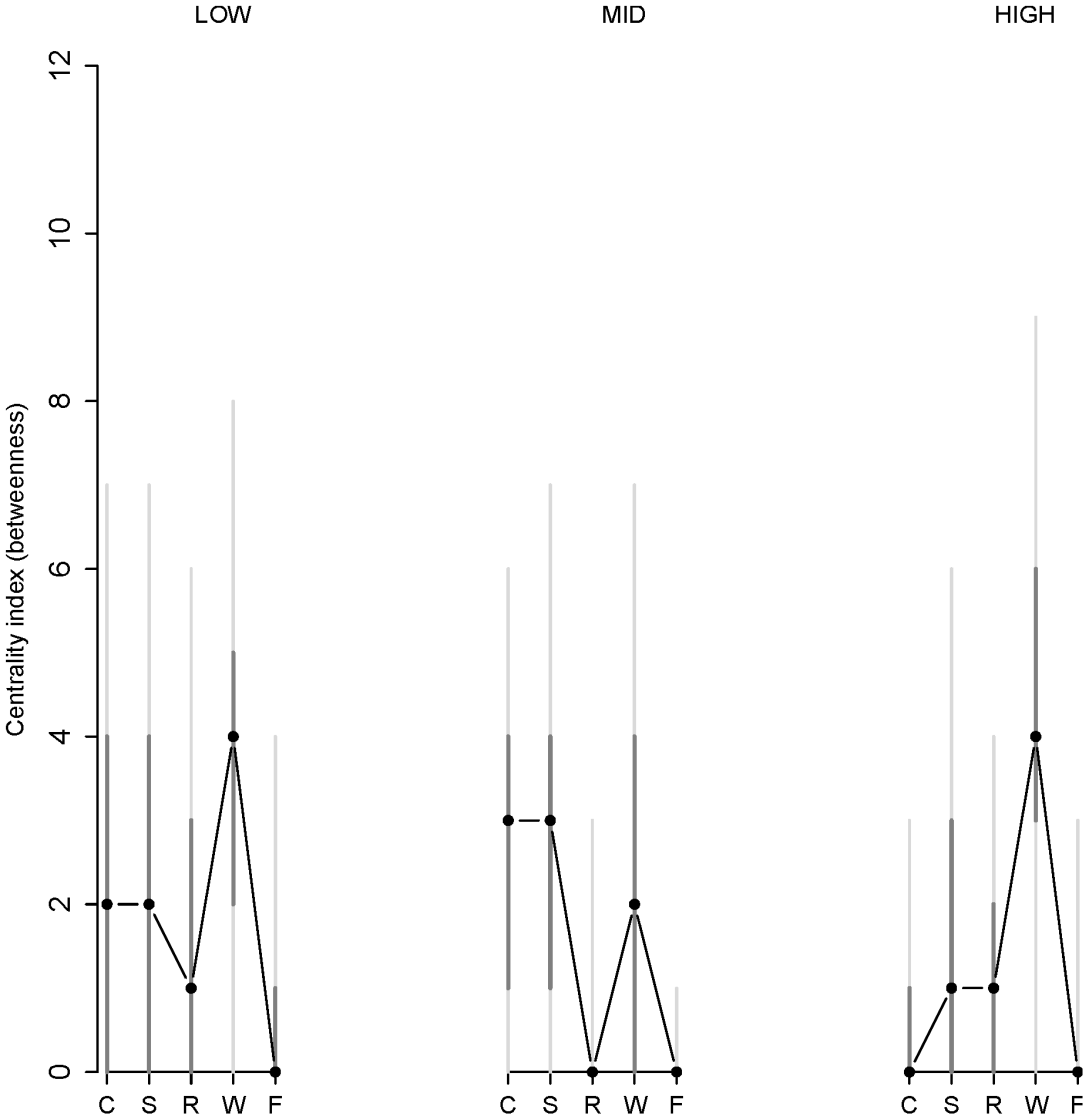
Centrality (betweenness) of each item in the network as a function of level of neuroticism in the validation dataset. Low, mid, and high neuroticism are shown from left to right. The labels of the items are abbreviated by their first letter (C = cheerful, S = sad, R = relaxed, W = worry and F = fearful). The black dots are the model-based estimate of betweenness, the darkgrey vertical lines represent 50% confidence intervals and the light grey vertical lines represent 95% confidence intervals (as estimated from the bootstrap method). Together, the median, 50% and 95% confidence intervals give information on how the node centrality for every item in all three networks is distributed.

In Figure 6 the results of the betweenness centrality analysis for low, middle and high neuroticism of the validation dataset are shown. These results can be compared with Figure 4, since the results of the main dataset with five variables are very similar to those with six variables (see Appendix S3 for the betweenness centrality figure of the main dataset with only five variables). Although worry is again one of the most central nodes in the high neuroticism group, there is no clear shift in centrality between the groups, which we found in the main dataset (see Figure 4). In fact, worry seems to be also one of the most central nodes in the low and mid neuroticism group in this dataset. The difference in centrality between the datasets could be related to the overall level of neuroticism. After applying a linear transformation to approximately equate the neuroticism measures in the two groups, we found that in the main dataset the average neuroticism score (*M = *40.7; *SD* = 7.4) was markedly higher than in the validation set (*M = *31.1; *SD* = 12.1; *t* (148.68)* = −*6.9, *p*<0.0001). Furthermore, as noted in the Method section, worry was assessed slightly differently in the two datasets, which could also account for the difference in the centrality of worry.

## Discussion

In this paper, we have presented a combination of vector autoregressive (VAR) modeling and multilevel modeling which, to the best of our knowledge, is the first method suited for inferring networks from ESM data. The modeling technique combines time series with individual differences. This strategy allows us to cope with the peculiarities of ESM data (e.g., short time series, significant individual differences) but also opens up unique possibilities for studying individual differences in dynamic structure. Thus, the methodology is an important addition to network methodologies that are currently being developed in personality and clinical psychology and psychiatry [2], [3]. For simplicity, we limited the analysis to six variables in this paper, but in principle the analysis is generalizable to larger datasets and to different time series models (e.g., models with different lags). Thus, the methodology is sufficiently flexible to give rise to a relatively comprehensive approach. Furthermore, it is a great advantage that such complex dynamics between several variables can be easily visualized as a network with the *R* package *qgraph* [46]. Illustrating the dynamical interaction between several variables helps to give an immediate intuitive understanding of the complex structure of the model, and is more insightful than a mere verbal explanation [78], [79].

The multilevel-VAR method combines a nomothetic approach, which makes it possible to generalize findings to a population level, with an idiographic approach, which models dynamical processes at the level of the individual person. In our study, for instance, the fixed effects of the model form a plausible network at group level, which shows the average dynamics between six mood related variables at baseline. Importantly, this population network was replicated in the validation dataset. In both datasets the same dynamics between the variables were found, supporting the validity of the multilevel-VAR method.

In addition, individual heterogeneity can be easily assessed using the random effects estimated in the model. Again, a similar network of individual heterogeneity was found in the validation data. Although some links in the networks of individual heterogeneity differed between the two datasets, the majority of them showed a similar degree of individual variability. Because the two datasets contain different populations, it is to be expected that not all links show the same amount of individual heterogeneity. Intra-individual time series can also be studied by combining fixed and random effects for each subject, which results in individual networks. Thus, our method successfully combines nomothetic and idiographic approaches to data analysis.

In time, the latter approach may lead to improved understanding of intra-individual functioning; this may in turn lead to better therapeutic interventions. A network analysis of a subject receiving therapy may show, for example, that the link between rumination and sadness is the strongest link in the network and that a therapy should intervene on that link to improve the overall mood.

In addition to the visualization of the multilevel-VAR analysis, the inferred networks open a range of new questions and possibilities that arise from network theory, and thus open a whole new research field. On the one hand, the local structure or specific connections can be studied with a local network analysis; on the other hand, the overall structure of the network can be studied with a global network analysis.

An example of a network analysis is node centrality as assessed through a betweenness measure. With this global network analysis we identified the most central node in a network for three groups with a different neuroticism level (low, mid and high). Our results revealed that in general, the node ‘worrying’ was more central in the high neuroticism group than in the low or mid neuroticism group. This could be interpreted as indicating that worrying in general has a greater influence on the network in the high neuroticism group than in the low and mid neuroticism group. In the validation dataset there was no clear shift in worry in the high neuroticism group compared to the low and mid neuroticism group, but in this study worry was assessed slightly differently than in the main study, and furthermore, the population was different (college students instead of older subjects with residual depressive symptoms); in general, the subjects had lower neuroticism scores. More research is needed to study the relation between neuroticism and node centrality in different kinds of populations.

Future research may focus on developing similar local and global network analyses, specifically suited for networks inferred from ESM data, and on evaluating the implications of these results. Thus, the presented methodology enables the use of network approaches in clinical research and open new possibilities to analyze and understand the structure of disorders, not only by inferring and visualizing the interaction between the variables, but also by further analyzing the new inferred networks.

## Supporting Information

Appendix S1 **This file contains the software code necessary to perform the analyses that result in the main figures reported in this article.**
(R) Click here for additional data file.

Appendix S2 **This file contains a more elaborate description of the pseudo-likelihood method.**
(PDF)

Appendix S3 **This file contains all figures of the replication study.**
(PDF)

Data S1 **This file contains the data necessary to perform the analyses that result in the main figures reported in this article.**
(TXT)

## References

[1] Borsboom D (2008) Psychometric perspectives on diagnostic systems. J Clin Psychol 64: 1089–1108 doi: 10.1002/jclp.20503 18683856 10.1002/jclp.20503

[2] Borsboom D, Cramer AOJ, Schmittmann VD, Epskamp S, Waldorp LJ (2011) The small world of psychopathology. PLoS One 6: e27407 doi: 101371/journal.pone.0027407 22114671 10.1371/journal.pone.0027407 PMC3219664

[3] Cramer AOJ, Waldorp LJ, van der Maas HLJ, Borsboom D (2010) Comorbidity: A network perspective. Behav Brain Sci 33: 137–193 doi:10.1017/S0140525X09991567 20584369 10.1017/S0140525X09991567

[4] Cramer AOJ, Borsboom D, Aggen SH, Kendler KS (2012) The pathoplasticity of dysphoric episodes: Differential impact of stressful life events on the pattern of depressive symptom inter-correlations. Psychol Med 42: 957–967 doi:10.1017/S003329171100211X 22093641 10.1017/S003329171100211X PMC3315770

[5] Kendler KS (2012) Levels of explanation in psychiatric and substance use disorders: Implications for the development of an etiologically based nosology. Mol Psychiatry 17: 11–21 doi:10.1038/mp.2011.70 21670729 10.1038/mp.2011.70 PMC3215837

[6] Schmittmann VD, Cramer AOJ, Waldorp LJ, Epskamp S, Kievit RA, et al.. (2011) Deconstructing the construct: A network perspective on psychological phenomena. New Ideas Psychol. doi: 10.1016/j.newideapsych.2011.02.007.

[7] Kendler KS, Zachar P, Craver C (2011) What kinds of things are psychiatric disorders? Psychol Med 41: 1143–1150 doi:10.1017/S0033291710001844 20860872 10.1017/S0033291710001844

[8] Ebner-Priemer UW, Eid M, Kleindienst N, Stabenow S, Trull TJ (2009) Analytic strategies for understanding affective (in)stability and other dynamic processes in psychopathology. J Abnorm Psychol 118: 195–202 doi: 10.1037/a0017075 19222325 10.1037/a0014868

[9] Kuppens P, Oravecz Z, Tuerlinckx F (2010) Feelings change: Accounting for individual differences in the temporal dynamics of affect. J Pers Soc Psychol 99: 1042–1060 doi: 10.1037/a0020962 20853980 10.1037/a0020962

[10] Meney I, Waterhouse J, Atkinson G, Reilly T, Davenne D (1998) The effect of one night’s sleep deprivation on temperature, mood, and physical performance in subjects with different amounts of habitual physical activity. Chronobiol Int 14: 125–32. 10.3109/07420529808998695 9706412

[11] Geschwind N, Peeters F, Drukker M, van Os J, Wichers M (2011) Mindfulness training increases momentary positive emotions and reward experience in adults vulnerable to depression: A randomized controlled trial. J Consult Clin Psychol 79: 618–628 doi: 10.1037/a002459 21767001 10.1037/a0024595

[12] Csikszentmihalyi M, Larson R (1987) Validity and reliability of the experience-sampling method. J Nerv Ment Dis 175: 526–536. 3655778 10.1097/00005053-198709000-00004

[13] Stone AA, Shiffman SS (1994) Ecological momentary assessment (EMA) in behavioral medicine. Ann Behav Med 16: 199–202.

[14] Bolger N, Davis A, Rafaeli E (2003) Diary methods: Capturing life as it is lived. Annu Rev Psychol 54: 579–616 doi: 10.1146/annurev.psych.54.101601.145030 12499517 10.1146/annurev.psych.54.101601.145030

[15] Hendrickx DM, Hendriks MMWB, Eilers PHC, Smilde AK, Hoefsloot HCJ (2011) Reverse engineering of metabolic networks: A critical assessment. Mol Biosyst 7: 511–520 doi: 10.1039/c0mb00083c 21069230 10.1039/c0mb00083c

[16] Sporns O (2011) Networks of the Brain. Cambridge, MA: MIT Press.

[17] Gates KM, Molenaar PC (2012) Group search algorithm recovers effective connectivity maps for individuals in homogeneous and heterogeneous samples. Neuroimage 63: 310–319 doi: 10.1016/j.neuroimage.2012.06.026 22732562 10.1016/j.neuroimage.2012.06.026

[18] Hamilton JD (1994) Time Series Analysis. Princeton: Princeton University Press.

[19] Shumway RS, Stoffer DS (2006) Time series analysis and its applications: With R examples. New York: Springer.

[20] Schwartz JE, Stone AA (1998) Strategies for analysing ecological momentary assessment data. Health Psychol 17: 6–16. 9459065 10.1037//0278-6133.17.1.6

[21] The *R* project for statistical computing. Available: http://www.Rproject. Accessed 2012 Dec 12.

[22] Barrett LF (1998) Discrete emotions or dimensions? The role of valence focus and arousal focus. Cogn Emot 12: 579–599.

[23] Reisenzein R (1994) Pleasure-arousal theory and the intensity of emotions. J Pers Soc Psychol 67: 525–539.

[24] Russell JA (1980) A circumplex model of affect. J Pers Soc Psychol 39: 1161–1178.

[25] Russell JA, Weiss A, Mendelsohn GA (1989) Affect grid: A single-item scale of pleasure and arousal. J Pers Soc Psychol 57: 493–502.

[26] Smith CA, Ellsworth PC (1985) Patterns of cognitive appraisal in emotion. J Pers Soc Psychol 48: 813–838. 3886875

[27] Baas M, De Dreu CKW, Nijstad BA (2008) A meta-analysis of 25 years of mood-creativity research: Hedonic tone, activation, or regulatory focus? Psychol Bull 134: 779–806 doi: 10.1037/a0012815 18954157 10.1037/a0012815

[28] Larsen RJ, Diener E (1992) Promises and problems with the circumplex model of emotion. In: Clark MS, editor. Review of personality and social psychology (Vol. 13): Emotion. Newbury Park, CA: Sage. 25–59.

[29] Watson D, Tellegen A (1985) Toward a consensual structure of mood. Psychol Bull 98: 219–235. 3901060 10.1037//0033-2909.98.2.219

[30] Brosschot JF, Gerin W, Thayer JF (2006) The perseverative cognition hypothesis: A review of worry, prolonged stress-related physiological activation, and health. J Psychosom Res 60: 113–124 doi:10.1016/j.jpsychores.2005.06.074 16439263 10.1016/j.jpsychores.2005.06.074

[31] Borkovec TD, Ray WJ, Stöber J (1998) Worry: A cognitive phenomenon intimately linked to affective, physiological, and interpersonal behavioral processes. Cognit Ther Res 22: 561–576.

[32] Gruber J, Eidelman P, Harvey AG (2008) Transdiagnostic emotion regulation processes in bipolar disorder and insomnia. Behav Res Ther 46: 1096–1100 doi:10.1016/j.brat.2008.05.004 18684436 10.1016/j.brat.2008.05.004

[33] Pfaff B (2008) Analysis of Integrated and Cointegrated Time Series with R. New York: Springer.

[34] Snijders T, Bosker R (2012) Multilevel analysis: An introduction to basic and advanced multilevel modeling. London: Sage Publications.

[35] Walls TA, Schafer JL (2006) Models for Intensive Longitudinal Data. Oxford: Oxford University Press.

[36] Lodewyckx T, Tuerlinckx F, Kuppens P, Allen NB, Sheeber LB (2011) A hierarchical state space approach to affective dynamics. J Math Psychol 55: 68–83 doi:10.1016/j.jmp.2010.08.004 21516216 10.1016/j.jmp.2010.08.004 PMC3079909

[37] Oravecz Z, Vandekerckhove J, Tuerlinckx F (2011) A hierarchical latent stochastic differential equation model for affective dynamics. Psychol Methods 16: 468–490 doi: 10.1037/a0024375 21823796 10.1037/a0024375

[38] Pe ML, Kuppens P (2012) The dynamic interplay between emotions in daily life: Augmentation, blunting, and the role of appraisal overlap. Emotion 12: 1320–1328 doi: 10.1037/a0028262 22642355 10.1037/a0028262

[39] Tschacher W, Ramseyer F (2009) Modeling psychotherapy process by time-series panel analysis (TSPA). Psychother Res 19: 469–481 doi: 10.1080/10503300802654496 19585371 10.1080/10503300802654496

[40] Tschacher W, Zorn P, Ramseyer F (2012) Change Mechanisms of Schema-Centered Group Psychotherapy with Personality Disorder Patients. PLoS ONE 7: e39687 doi:10.1371/journal.pone.0039687 22745811 10.1371/journal.pone.0039687 PMC3382158

[41] Schmid CH (2001) Marginal and dynamic regression models for longitudinal data. Stat Med 20: 3295–3311 doi:10.1002/sim.950 11746319 10.1002/sim.950

[42] Funatogawa I, Funatogawa T, Ohashi Y (2007) An autoregressive linear mixed effects model for the analysis of longitudinal data which show profiles approaching asymptotes. Stat Med 26: 2113–2130 doi: 10.1002/sim.2670 16900564 10.1002/sim.2670

[43] Horváth C, Wieringa JE (2008) Pooling data for the analysis of dynamic marketing systems. Stat Neerl 62: 208–229 doi:10.1111/j.1467-9574.2007.00382.x

[44] Boccaletti S, Latora V, Moreno Y, Chavez M, Hwang DU (2006) Complex networks: Structure and dynamics. Phys Rep 424: 175–308 doi:10.1016/j.physrep.2005.10.009

[45] Benjamini Y, Hochberg Y (1995) Controlling the false discovery rate: A practical and powerful approach to multiple testing. J R Stat Soc Series B Stat Methodol 57: 289–300.

[46] Epskamp S, Cramer AOJ, Waldorp LJ, Schmittmann VD, Borsboom D (2012) Qgraph: Network visualizations of relationships in psychometric data. J Stat Softw 48: 1–18.

[47] Arnold B, Strauss D (1991) Pseudolikelihood estimation: Some examples. Sankhya Ser B 53: 233–243.

[48] Fieuws S, Verbeke G (2006) Pairwise fitting of mixed models for the joint modeling of multivariate longitudinal profiles. Biometrics 62: 424–431 doi: 10.1111/j.1541-0420.2006.00507.x 16918906 10.1111/j.1541-0420.2006.00507.x

[49] Bates DM, Maechler M, Bolker B (2011) lme4: Linear mixed-effects models using S4 classes. R package version 0.999999-0.

[50] Verbeke G, Molenberghs G (2000) Linear mixed models for longitudinal data. New York: Springer.

[51] Baltagi BH (2005) Economotric analysis of panel data. Chichester: Wiley.

[52] Verbeke G, Spiessens B, Lesaffre E (2001) Conditional linear mixed models. Am Stat 55: 25–34 doi:10.1198/000313001300339905

[53] Neyman J, Scott E (1948) Consistent estimates based on partially consistent observations. Econometrica 16: 1–32.

[54] Gelman A, Hill J (2007) Data Analysis Using Regression and Multilevel/Hierachical Models. Cambridge: Cambridge University Press.

[55] Tuerlinckx F, Rijmen F, Verbeke G, De Boeck P (2006) Statistical inference in generalized linear mixed models: A review. Br J Math Stat Psychol 59: 225–255 doi:10.1348/000711005X79857 17067411 10.1348/000711005X79857

[56] Opsahl T, Agneessens F, Skvoretz F (2010) Node centrality in weighted networks: Generalizing degree and shortest paths. Soc Networks 32: 245–251 doi:10.1016/j.socnet.2010.03.006

[57] Hoekstra HA, Ormel J, De Fruyt F (1996) NEO PI-R. NEO FFI. Big Five Persoonlijkheidsvragenlijsten: Handleiding [NEO PI-R. NEO FFI. Big Five Personality questionaires: Manual]. Lisse: Swets & Zeitlinger.

[58] Efron B, Tibshirani R (1994) An Introduction to the Bootstrap. New York: Chapman & Hall.

[59] Pe ML, Raes F, Koval P, Brans K, Verduyn P, et al.. (2012) Interference resolution moderates the impact of rumination and reappraisal on affective experiences in daily life. Cogn Emot. In press. doi:10.1080/02699931.2012.719489. 10.1080/02699931.2012.719489 22966838

[60] Pe ML, Koval P, Kuppens P (2012) Executive well-being: Updating of positive stimuli in working memory is associated with subjective well-being. Cognition. In Press. doi:10.1016/j.cognition.2012.10.002. 10.1016/j.cognition.2012.10.002 23122635

[61] Koval P, Kuppens P, Allen NB, Sheeber LB (2012) Getting stuck in depression: The roles of rumination and emotional inertia. Cogn Emot 26: 1412–1427. 22671768 10.1080/02699931.2012.667392

[62] Gosling S, Rentfrow P, Swann W (2003) A very brief measure of the big five personality domains. J Res Pers 37: 504–528 doi:10.1016/S0092-6566(03)00046-1

[63] Hofmans J, Kuppens P, Allik J (2008) Is short in length short in content? An examination of the domain representation of the Ten Item Personality Inventory scales in Dutch language. Pers Individ Dif 45: 750–755 doi:10.1016/j.paid.2008.08.004

[64] Lavie P (2001) Sleep-wake as a biological rhythm. Annu Rev Psychol 52: 277–303. 11148307 10.1146/annurev.psych.52.1.277

[65] Box JEP, Jenkins GM, Reinsel GC (1994) Time series analysis: Forecasting and control. Englewood Cliffs, NJ: Prentice Hall.

[66] Kwiatkowski D, Phillips PCB, Schmidt P (1992) ShinY (1992) Testing the null hypothesis of stationarity against the alternative of a unit root. J Econom 54: 159–178.

[67] Schwarz G (1978) Estimating the dimension of a model. Annals of Statistics 6: 461–64.

[68] Lütkepohl H (1993) Introduction to Multiple Time Series Analysis. Berlin: Springer.

[69] Fredrickson BL, Joiner T (2002) Positive emotions trigger upward spirals toward emotional well-being. Psychol Sci 13: 172–75. 11934003 10.1111/1467-9280.00431

[70] Zelenski JM, Larsen RJ (2000) The distribution of basic emotions in everyday life: A state and trait perspective from experience sampling data. J Res Pers 34: 178–197 doi:10.1006/jrpe.1999.2275

[71] Larsen JT, McGraw AP, Cacioppo JT (2001) Can people feel happy and sad at the same time? J Pers Soc Psychol 81: 684–696 doi: 10.1037//0022-3514.81.4.684 11642354

[72] Russell JA, Carroll JM (1999) On the bipolarity of positive and negative affect. Psychol Bull 125: 3–30. 9990843 10.1037/0033-2909.125.1.3

[73] Watson D, Wiese D, Vaidya J, Tellegen A (1999) The two general activation systems of affect: Structural findings, evolutionary considerations, and psychobiological evidence. J Pers Soc Psychol 76: 820–838.

[74] McLaughlin K, Borkovec TD, Sibrava NJ (2007) The effect of worry and rumination on affective states and cognitive activity. Behav Ther 38: 23–38. 17292692 10.1016/j.beth.2006.03.003

[75] Segerstrom SC, Tsao JCI, Alden LE, Craske MG (2000) Worry and rumination: Repetitive thought as a concomitant and predictor of negative mood. Cognit Ther Res 24: 671–688.

[76] Moberly NJ, Watkins ER (2008) Ruminative self-focus and negative affect: An experience sampling study. J Abnorm Psycho 117: 314–323 doi: 10.1037/0021-843X.117.2.314 10.1037/0021-843X.117.2.314 PMC2672047 18489207

[77] Muris P, Roelofs J, Meesters C, Boomsma P (2004) Rumination and worry in nonclinical adolescents. Cognit Ther Res 28: 539–554.

[78] Wild B, Eichler M, Friederich H, Hartmann M, Zipfel S, et al. (2010) A graphical vector autoregressive modelling approach to the analysis of electronic diary data. BMC Med Res Methodol 10: 28. Available: http://www.biomedcentral.com/1471-2288/10/28. Accessed 21 February 2012. 10.1186/1471-2288-10-28 PMC2869334 20359333

[79] Gather U, Imhoff M, Fried R (2002) Graphical models for multivariate time series from intensive care monitoring. Stat Med 21: 2685–2701 doi: 10.1002/sim.1209 12228885 10.1002/sim.1209

