# Correction: A Network Approach to Psychopathology: New Insights into Clinical Longitudinal Data

**DOI:** 10.1371/journal.pone.0096588

**Published:** 2014-04-24

**Authors:** The PLOS ONE Staff

Figure 4 and the bootstrap code are incorrect. In the R-code, included as a supplement to Bringmann et al. (2013), time dependency was not taken into account properly in the bootstrapping procedure. In the original analysis, the authors used a parametric bootstrap method. However, taking the time dependency properly into account is not easy and therefore they have opted for nonparametric bootstrapping procedure instead. In addition, they have implemented the bootstrap directly for the multilevel model (instead of the linear model as reported in the paper, which can only give approximate results because the original model was a multilevel model). As a result, the latter also implies that the R-code cannot be run on a standard computer, due to extra computational difficulty (bootstrapping large multilevel models is much more computationally demanding than bootstrapping linear models). Below the authors first present the corrected Figure 4 and the corrected Appendix S1 that features the new R-code. As can be seen from the corrected figure, the results are very similar as the ones reported in the paper and the conclusions remain unaffected.

**Figure 4:**
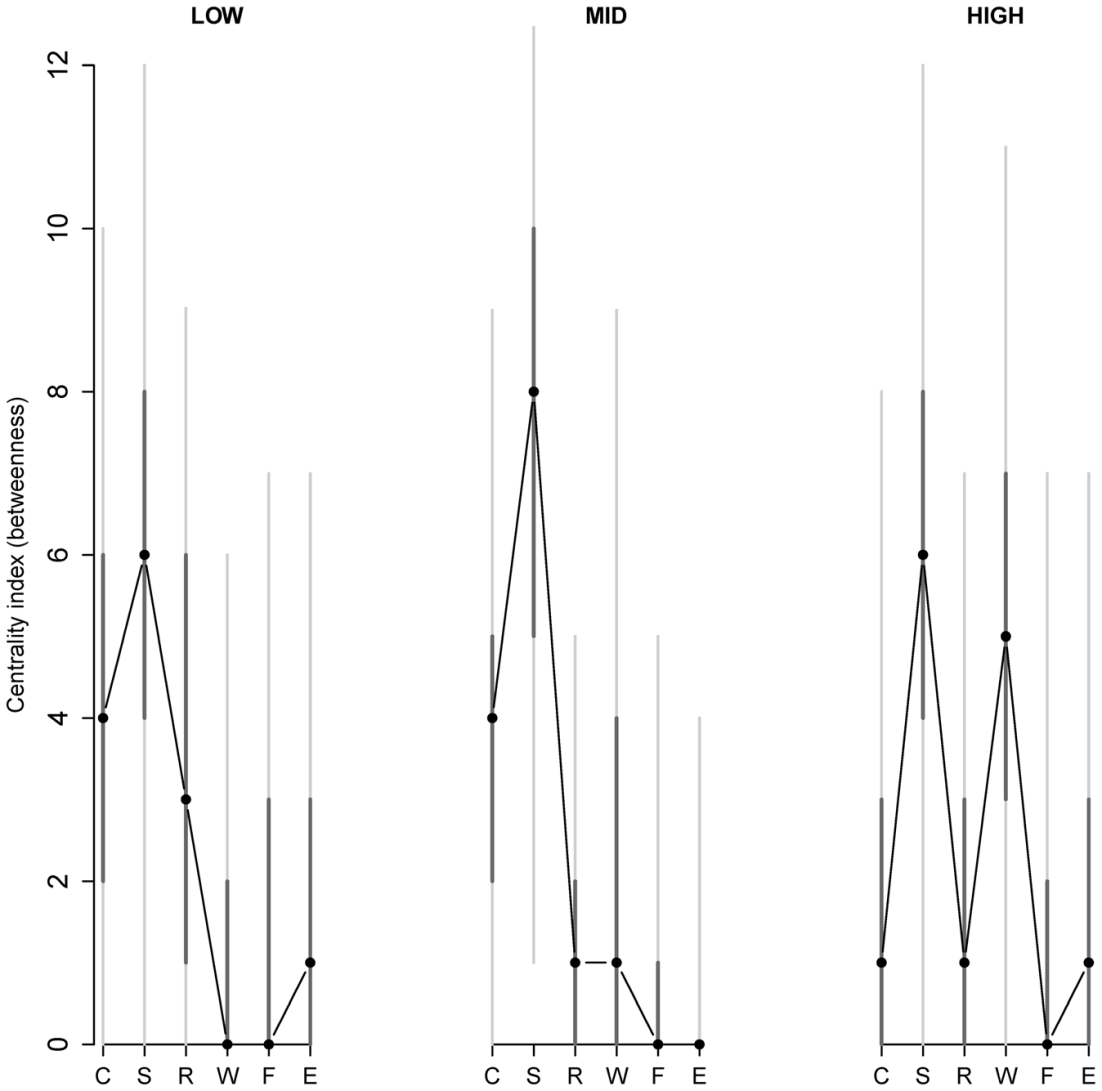
Centrality (betweenness) of each item in the network as a function of level of neuroticism at baseline. The centrality index (betweenness) of each item in the network as a function of level of neuroticism (low, mid, and high neuroticism are shown from left to right) at baseline are shown. The labels of the items are abbreviated by their first letter (C = cheerful, S = sad, R = relaxed, W =  worry, F = fearful). The black dots are the model-based estimates of betweenness, the dark grey vertical lines represent 50% confidence intervals and the light grey vertical lines represent 95% confidence intervals (as estimated by the bootstrap method). Together, the median, 50% and 95% confidence intervals give information on how the node centrality for every item in all three networks is distributed.

## Supporting Information

Appendix S1 This file contains the software code necessary to perform the analyses that result in the main figures reported in this article.
(R) Click here for additional data file.
