# Supplementary material for: A Network Approach to Psychopathology: New Insights into Clinical Longitudinal Data
Source: PLoS One. 2013 Apr 4;8(4):e60188. doi: 10.1371/journal.pone.0060188 (PMC3617177; doi:10.1371/journal.pone.0060188)
Supplement: Appendix S2 — This file contains a more elaborate description of the pseudo-likelihood method. (PDF) [file pone.0060188.s002.pdf]

Appendix S2: Pseudo-likelihood method and simulation study

Pseudo-likelihood method

Fitting a combination of VAR (for modeling the intra-individual change) and multilevel modeling (for modeling inter-individual differences) comes with certain difficulties. Therefore, we apply a model fitting procedure that is similar to estimating a traditional (i.e., non-multilevel) VAR by fitting a series of multiple regression models [1]. In our case, we fit a series of multilevel models, one for each item. Such an approach can be considered a specific case of the pseudo-likelihood method [2,3] in which not the likelihood itself is optimized to estimate the model's parameters, but rather an easier-to-calculate proxy to the likelihood (i.e., the pseudo-likelihood), which is constructed by considering a set of conditional and/or marginal densities. Our approach can be illustrated with a simple example (see also [2]): If one wants to estimate the parameters of a bivariate normal distribution (two means, two variances and a correlation) then one can estimate four out of five parameters (means and variances) by relying on the univariate marginals.

In this study, estimating the model's parameters is deferred to estimating the parameters from the marginal distributions of the six variables. As a result, the covariance matrix for random effects will not be estimated in a single step and not all of the covariance parameters will be estimated directly. Only eight-by-eight block matrices on the main diagonal from this general matrix pertaining to the same univariate multilevel analysis (i.e., $\mathrm{cov}(b_{kpj}, b_{kpj'})$) are estimated (there are six such block matrices). The remaining covariances in the 48-by-48 matrix (related to covariances between random effects of different univariate models, i.e. $\mathrm{cov}(b_{kpj}, b_{k'pj'})$) can be estimated in a subsequent step from the covariances between the predicted random effects. The error correlations, signifying the common disturbances to different variables, and the correlations between random effects of the different regression equations are not estimated in our approach. However, these parameters can be estimated in a second step by calculating the correlations between the level 1-residuals and level 2-residuals of the different univariate models. Relying on such an approach will probably lead to a small loss of efficiency compared to direct estimation.

We show by means of the simulations study, described in the next section, that using our approach, the point estimates of most directly and indirectly estimated parameters are on average close to the true values.

Simulation study

Goal

In order to investigate the performance of the multilevel-VAR model in recovering the network structure for the type of data used in this paper, we performed a simulation study. To optimize validity of the simulation study, we simulated data based on the parameter estimates obtained from the empirical study and fitted the data with the procedure outlined above and in the main text of this article. Specifically, we took the estimates based on the results of the items cheerful and worry.

As indicated above, we did not fit the multilevel-VAR model by fitting the multivariate model at once, but instead by fitting a series of univariate multilevel models. In these models, several of the parameters can be estimated directly (i.e., all fixed effects and random regression coefficients, variances and covariance of random effects parameters within one model), but some of the parameters could only indirectly be estimated (i.e., the covariances between errors and the covariances between random effects that are in different univariate models). Through this simulation study, we aimed to show that a pseudo-likelihood fitting of the multilevel-VAR model yields a reasonable approximation of all parameters.

Data simulation model

A multilevel-VAR model with random intercept and slopes was used for the simulation. For reasons of computational tractability, we have reduced, without loss of generalizability, the original six-variable multilevel-VAR model to a bivariate model. The model equations are (for $j = 1{,}2$; cheerful and worry respectively):

$$Y_{pdtj} = \gamma_{0pdj} + \gamma_{1pdj} \cdot Y_{p,d,t-1,1} + \gamma_{2pdj} \cdot Y_{p,d,t-1,2} + \varepsilon_{pdtj}, \qquad \text{(A1)}$$

where Y_{pdtj} represents the measurement for person p $(p = 1, \ldots 129)$ at day d $(d = 1, \ldots, 6)$ and time t of the j-th variable. In addition, it is assumed that the regression coefficients can be decomposed as follows (for $j = 1{,}2$), were β_{kj} represents the common effect of lagged variable k (for k=0, this is the intercept) on the dependent variable j, and b_{kpj} is the person-specific deviation of this general effect:

$$\gamma_{0pdj} = \beta_{0j} + b_{0pj}, \qquad \text{(A2)}$$

$$\gamma_{kpdj} = \beta_{kj} + b_{kpj}. \qquad \text{(A3)}$$

The intercepts (i.e., β_{01} and β_{02}) are set to 2.87 and 2.04, respectively. The other fixed effects parameters were fixed to $\beta_{11} = 0.28, \beta_{21} = -0.035, \beta_{12} = -0.048$ and $\beta_{22} = 0.26$, where, for example, β_{21}, stands for the effect of worry on cheerful. The two error terms ($\varepsilon_{pt1}, \varepsilon_{pt2}$) follow a bivariate normal distribution with mean vector zero, variances of $\sigma^2_{\varepsilon_1} = 1.3$ and $\sigma^2_{\varepsilon_2} = 1.56$ respectively and a correlation of 0.4. The two random intercept components (b_{0p1}, b_{0p2}) come from a bivariate normal population distribution with zero mean vector, variances 1.2 and 1.1 respectively and a correlation of 0.4. The four component vectors of random regression weights ($b_{1p1}, b_{2p1}, b_{1p2}, b_{2p2}$) are multivariate normally distributed with zero mean vector, variances (0.0169, 0.00810, 0.000784, 0.0256) and correlation of 0.4 among all pairs of components. Note that the random intercepts and random regression weights are independent.

Design of the simulation study

In our simulation study, we manipulated the number of time points as follows: *T*=20, 60, or 500. The number of participants was *N*=20, 129, or 500. We did not cross the factors, but instead, we started from the settings of the empirical example (i.e., *N*=129 and *T*=60) and then manipulated either the number of time points or the number of participants separately. In every condition, the number of simulated data sets (i.e., replications) was 500.

Results of the simulation study

In all figures, the left plot indicates the three different settings for the sample size and the right plot the three different setting of the number of time-points. All the fixed effects regression coefficients (i.e., the β's referring to intercepts and regression weights) were estimated very accurately with all different settings (Figure A1 and A2). The variance of the errors and the variance of all person specific regression weights (i.e., the *b*'s) including the intercept, are shown in figures A3-A5. From these plots, it can be seen that true point estimations of these parameters are accurate, often with less subjects or time points than used in the empirical study.

Figures A6 and A7 shows how accurately the correlations between parameters of the models were estimated in an indirect way. This was done for the error correlation between the models (i.e., the correlation between: $\varepsilon_{\text{pdt1}}$ and $\varepsilon_{\text{pdt2}}$), the random effects within one model

(i.e., the correlation between:b_{1p1} and b_{2p1}), and the random effects between the two models (i.e., the correlation between:b_{0p1} and b_{0p2}; b_{1p1} and b_{1p2}; b_{1p1} and b_{2p2}; b_{2p1} and b_{1p2}; b_{2p1} and b_{2p2}). Figure A6 shows that although in the first model the correlation between the two random effects of the betas could be estimated quite accurately with 60 time points and 129 subjects, in the second model the correlation between the random effects was estimated less accurately with 60 time points and 129 subjects than with more time points or subjects. There was also an estimation bias when the correlation of random effects (b's) between the models was estimated (Figure A7). However, the correlation of the error variances and random intercepts was estimated highly accurately, also between the two models (Figure A6). Thus, the random effects, except the random intercepts, were more difficult to estimate accurately and more subjects or time points are needed in that case. However, the model accurately estimated all the parameters that are of immediate relevance for this study.

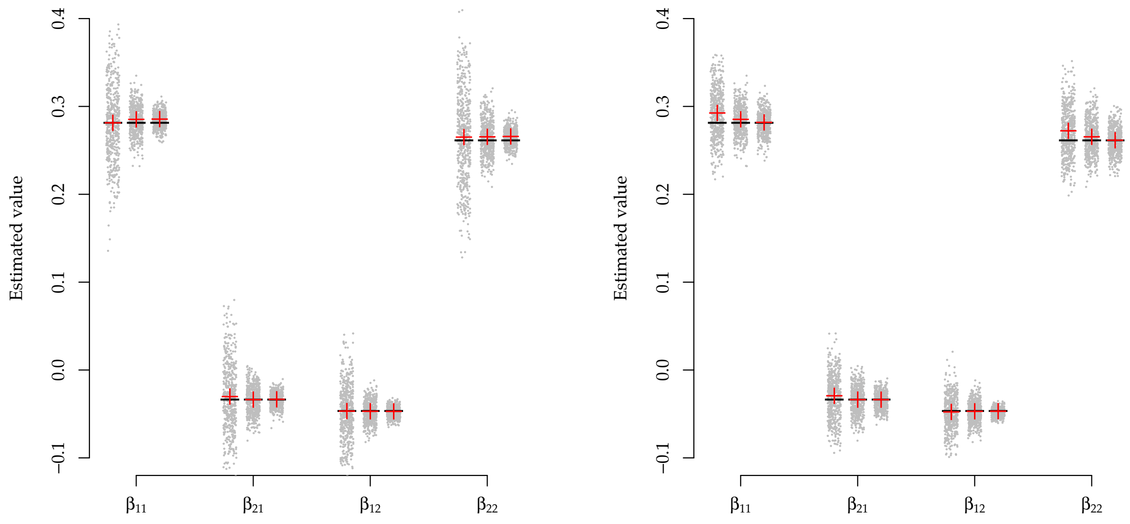

Figure A1. The recovery of the four average beta weights ($\beta_{11}, \beta_{21}, \beta_{12}, \beta_{22}$) for a varying number of participants (right panel, with T=60) and a varying number of time points (left panel, with N=129). The black line indicates the true value, and the red cross indicates the average estimate (from 500 replications). The grey dots are the 500 individual estimates (jittered along the x-axis for visual understanding). The middle condition is always the setting corresponding to the empirical example, with 60 time points and 129 participants.

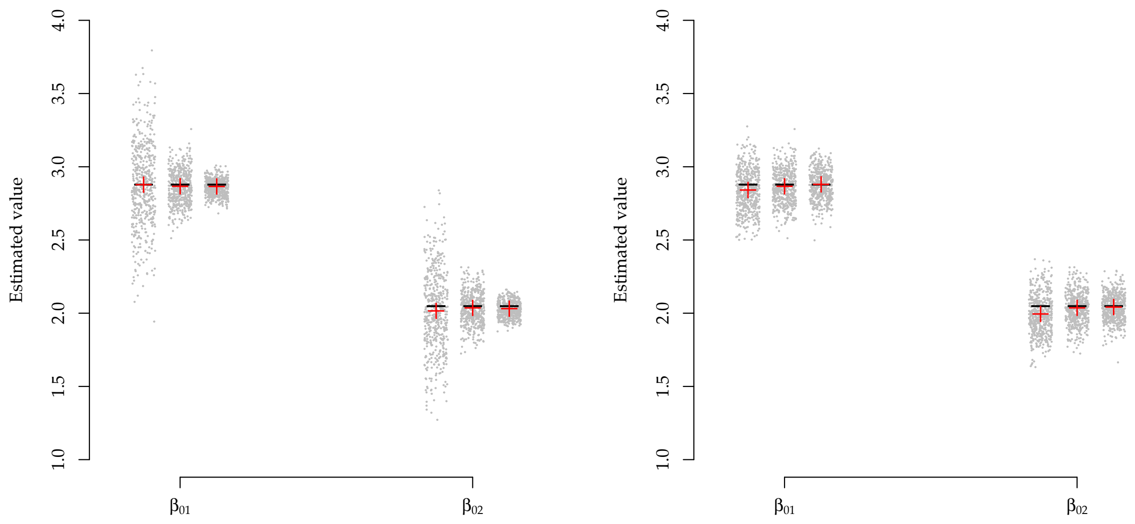

Figure A2. The recovery of the two average intercept coefficients (β_{01}, β_{02}) for a varying number of participants (right panel, with T=60) and a varying number of time points (left panel, with N=129). The black line indicates the true value, and the red cross indicates the average estimate (from 500 replications). The grey dots are the 500 individual estimates (jittered along the x-axis for visual understanding). The middle condition is always the setting corresponding to the empirical example, with 60 time points and 129 participants.

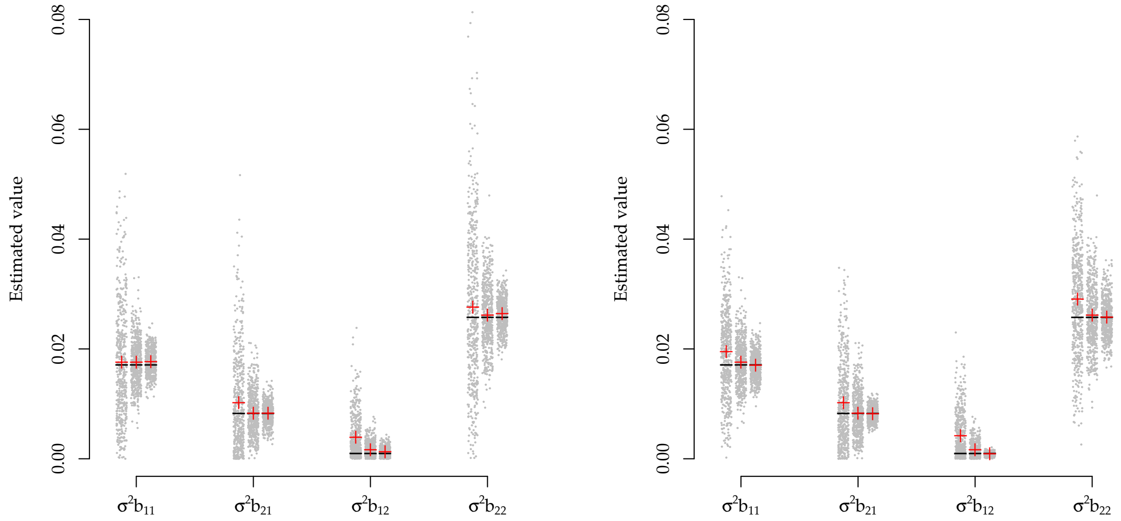

Figure A3. The recovery of the four variances of the person specific regression weights ($b_{11}, b_{21}, b_{12}, b_{22}$ see equation A3) for a varying number of participants (right panel, with T=60) and a varying number of time points (left panel, with N=129). The black line indicates the true value, and the red cross indicates the average estimate (from 500 replications). The grey dots are the 500 individual estimates (jittered along the x-axis for visual understanding). The middle condition is always the setting corresponding to the empirical example, with 60 time points and 129 participants.

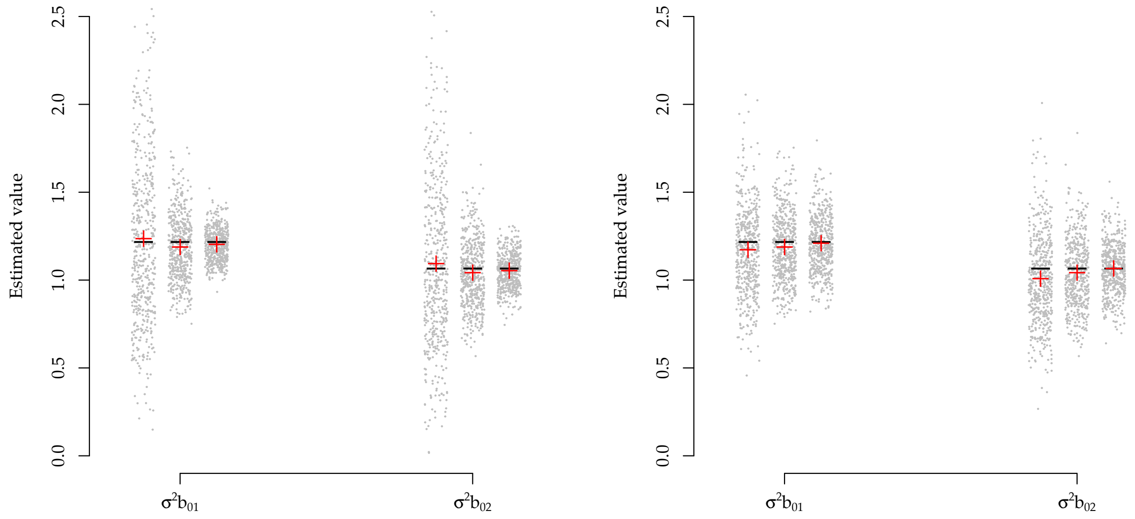

Figure A4. The recovery of the two variances of the person specific intercepts (b_{01}, b_{02}; see equation A2) for a varying number of participants (right panel, with T=60) and a varying number of time points (left panel, with N=129). The black line indicates the true value, and the red cross indicates the average estimate (from 500 replications). The grey dots are the 500 individual estimates (jittered along the x-axis for visual understanding). The middle condition is always the setting corresponding to the empirical example, with 60 time points and 129 participants.

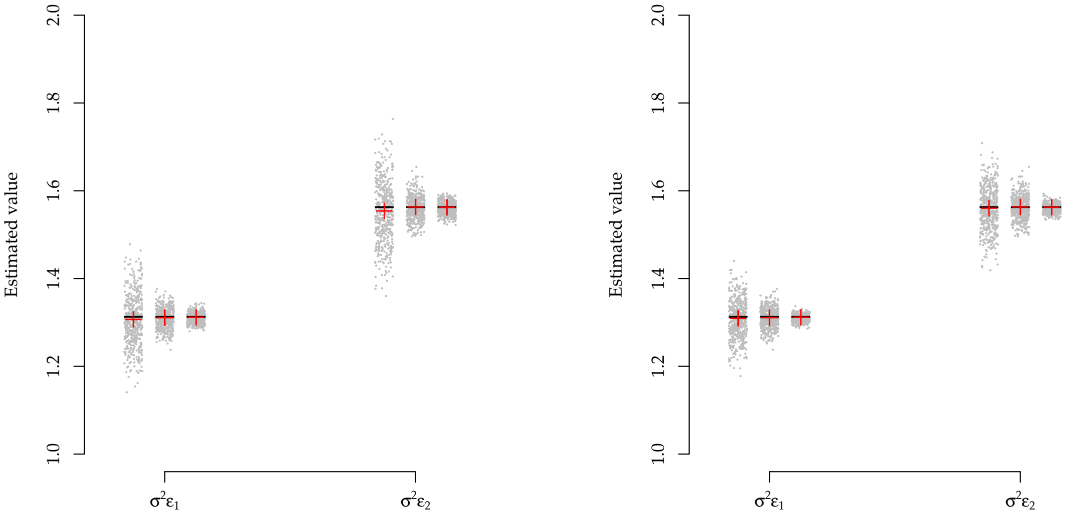

Figure A5. The recovery of the variances of the two error terms (ε_1 and ε_2) for a varying number of participants (right panel, with T=60) and a varying number of time points (left panel, with N=129). The black line indicates the true value, and the red cross indicates the average estimate (from 500 replications). The grey dots are the 500 individual estimates (jittered along the x-axis for visual understanding). The middle condition is always the setting corresponding to the empirical example, with 60 time points and 129 participants.

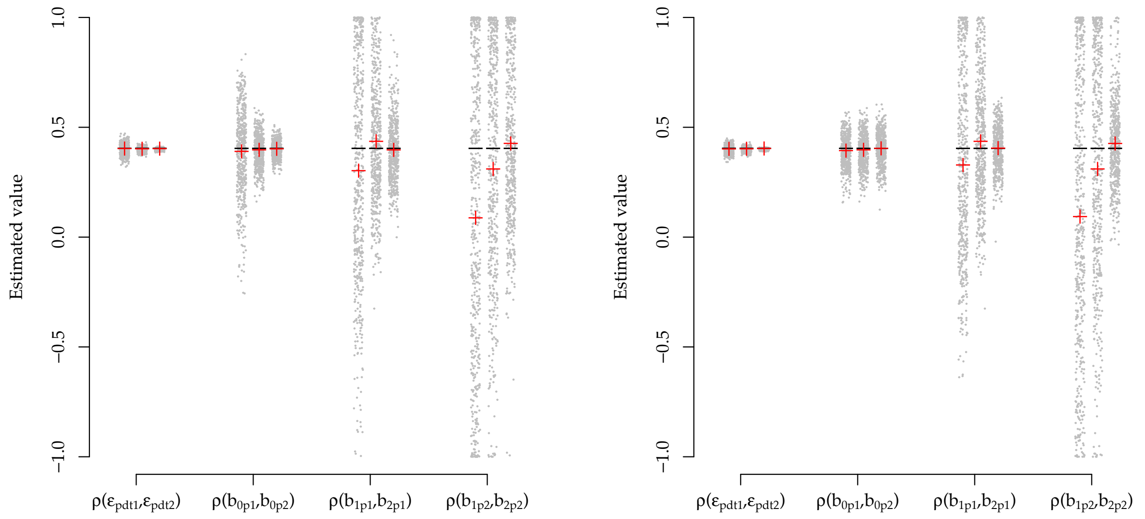

Figure A6. The recovery of the error correlations, the random intercept correlations and the correlations of the random effects within model 1 and 2, respectively. The simulation was done for a varying number of participants (right panel, with T=60) and a varying number of time points (left panel, with N=129). The black line indicates the true value, and the red cross indicates the average estimate (from 500 replications). The grey dots are the 500 individual estimates (jittered along the x-axis for visual understanding). The middle condition is always the setting corresponding to the empirical example, with 60 time points and 129 participants.

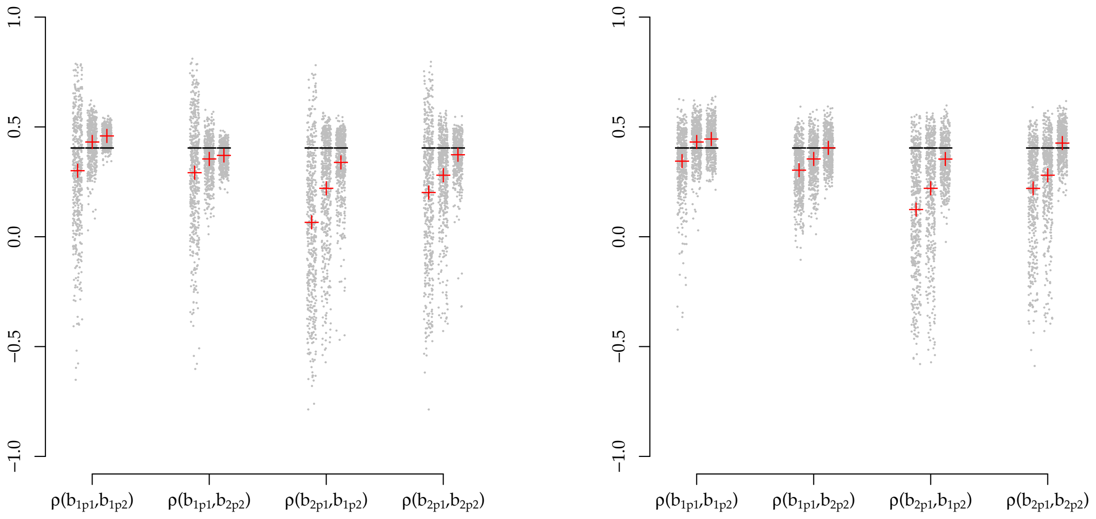

Figure A7. The recovery of correlations of random effects between the models of cheerful and worry. The simulation is done for a varying number of participants (right panel, with T=60) and a varying number of time points (left panel, with N=129). The black line indicates the true value, and the red cross indicates the average estimate (from 500 replications). The grey dots are the 500 individual estimates (jittered along the x-axis for visual understanding). The middle condition is always the setting corresponding to the empirical example, with 60 time points and 129 participants.

References

1. Hamilton JD (1994) Time series analysis. Princeton: Princeton University Press.

2. Arnold B, Strauss D (1991) Pseudolikelihood estimation: Some examples. Sankhya Ser B 53: 233-243.

3. Fieuws S, Verbeke G (2006) Pairwise fitting of mixed models for the joint modeling of multivariate longitudinal profiles. Biometrics 62: 424-431. doi: 10.1111/j.1541-0420.2006.00507.x.
