# Supplementary material for: A Network Approach to Psychopathology: New Insights into Clinical Longitudinal Data
Source: PLoS One. 2013 Apr 4;8(4):e60188. doi: 10.1371/journal.pone.0060188 (PMC3617177; doi:10.1371/journal.pone.0060188)
Supplement: Appendix S3 — This file contains all figures of the replication study. (PDF) [file pone.0060188.s003.pdf]

Appendix S3: Figures of the replication study

Population networks

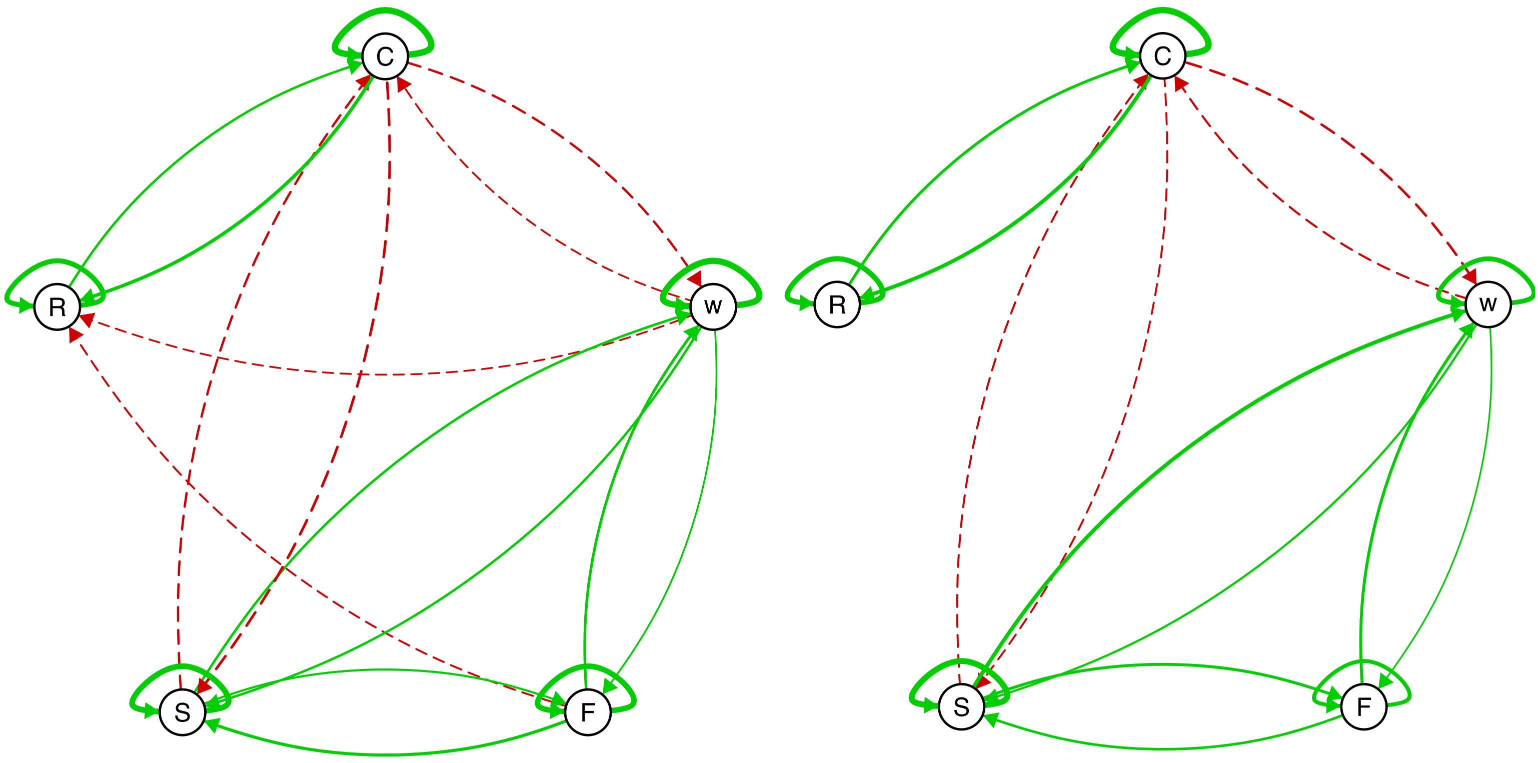

Figure A1. This figure shows the population network of the main data set (left panel) and the validation dataset (right panel) for five items (C=cheerful, W=worry, F=fearful, S=sad and R=relaxed). Solid green arrows correspond to positive arrows and red dashed arrows to negative connections. Only arrows that surpass the significance threshold are shown (i.e., for which the p-value of the t-statistic is smaller than 0.05). Arrows can be either red, indicating a negative relationship (i.e., $\beta_{kj}<0$), or green, indicating a positive relationship (i.e., $\beta_{kj}>0$). The two networks are almost identical (Pearson correlation of 0.95, see main text for more information). However, more links are significant in the main dataset and are shown in the figure than in the validation dataset (using FDR controlled at 5%). This is likely to be due to the fact that there are more subjects (129 vs. 97) and more time points (70 vs. 53) in the main dataset than in the validation dataset.

Inter-individual differences networks

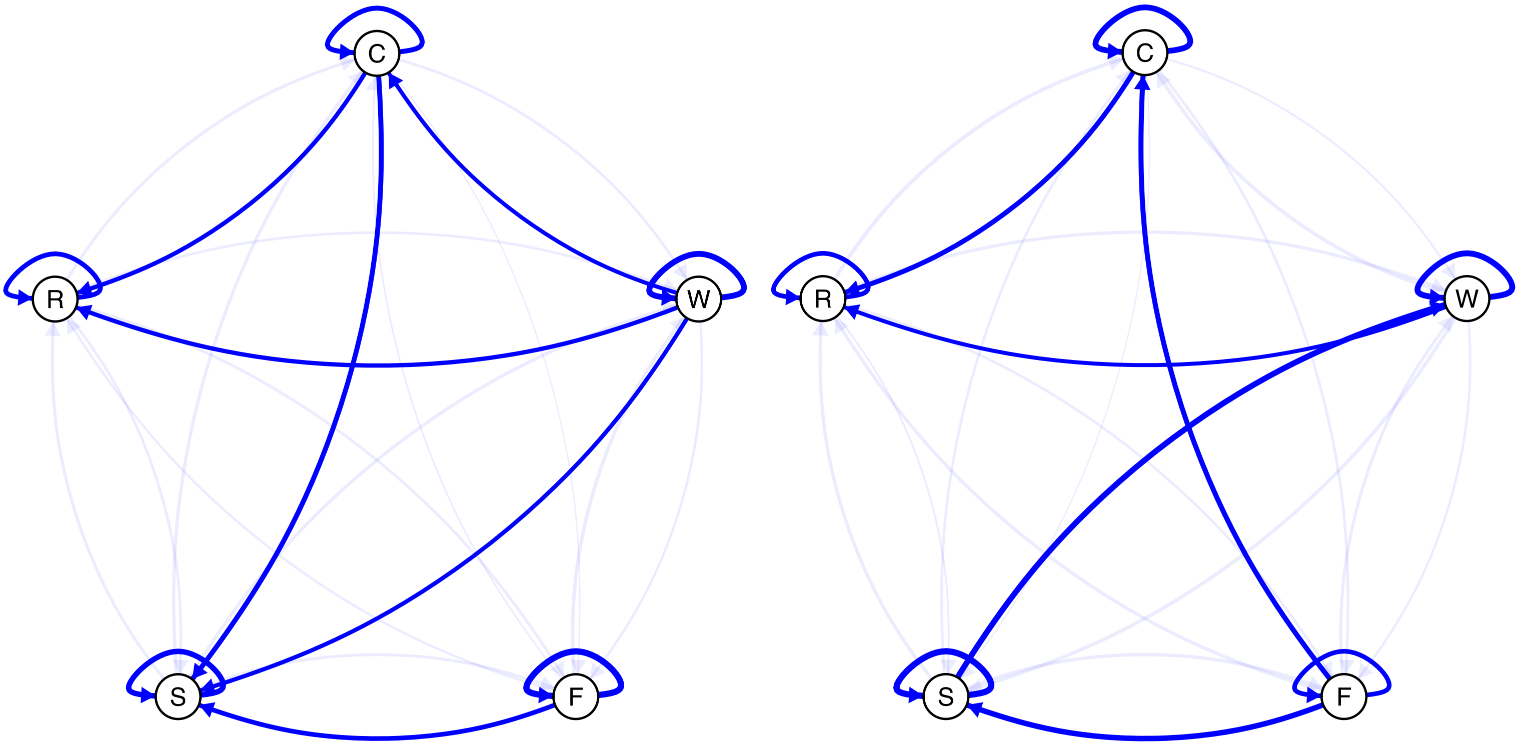

Figure A2. This figure shows the inter-individual network of the main dataset (left panel) and the validation dataset (right panel) for five items (C=cheerful, W=worry, F=fearful, S=sad and R=relaxed). The thickness of the arrows is based on the size of the standard deviation of the random effects. To construct the figure, we have put a cutoff of 0.1 on the standard deviation and only the standard deviations above the cutoff are shown with a non-transparent arrow.

Global network analysis: betweenness centrality

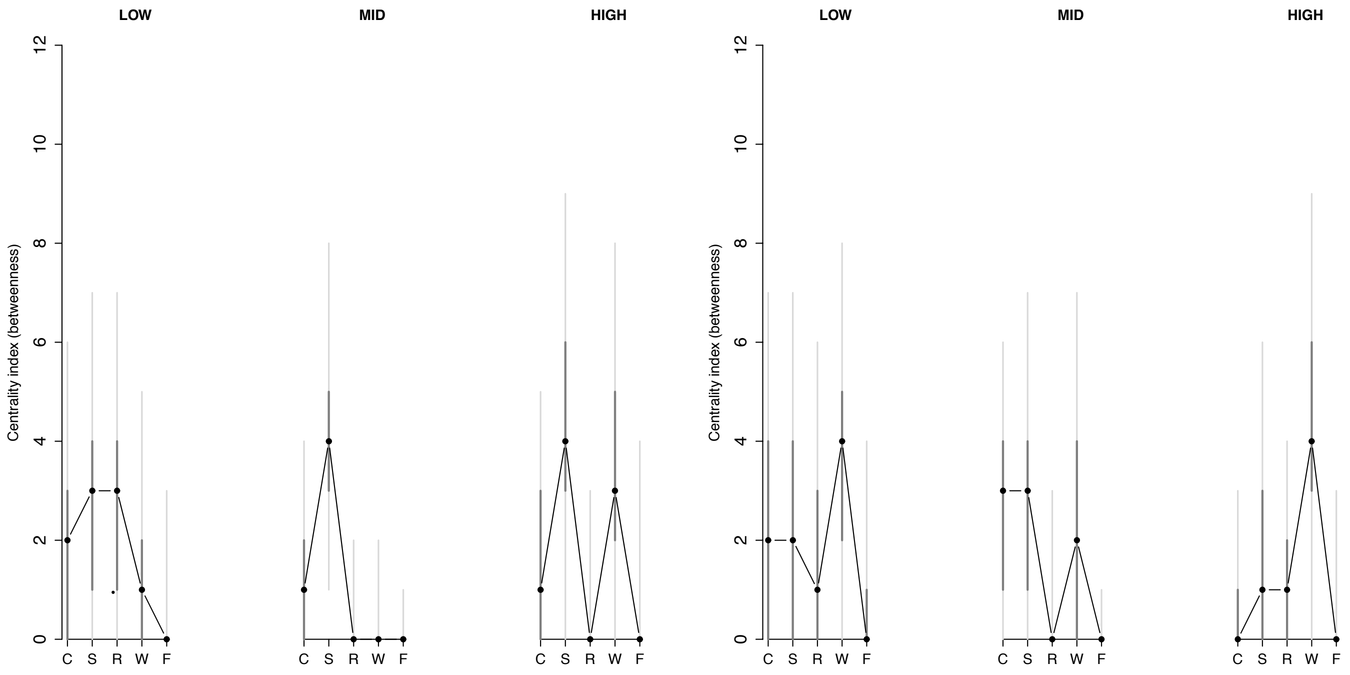

Figure A3. For both the main dataset (left panel) and the validation dataset (right panel) the centrality index (betweenness) of each item in the network as a function of level of neuroticism (low, mid, and high neuroticism are shown from left to right) at baseline are shown. The labels of the items are abbreviated by their first letter (C=cheerful, S=sad, R=relaxed, W= worry, F=fearful). The black dots are the model-based estimate of betweenness, the darkgrey vertical lines represent 50% confidence intervals and the light grey vertical lines represent 95% confidence intervals (as estimated from the bootstrap method). Together, the median, 50% and 95 % confidence intervals give information on how the node centrality for every item in all three networks is distributed.
